# A Transition Control Mechanism for Artificial Bee Colony (ABC) Algorithm

**DOI:** 10.1155/2019/5012313

**Published:** 2019-04-01

**Authors:** Selcuk Aslan

**Affiliations:** Department of Computer Engineering, Ondokuz Mayıs University, Samsun, Turkey

## Abstract

Artificial Bee Colony (ABC) algorithm inspired by the complex search and foraging behaviors of real honey bees is one of the most promising implementations of the Swarm Intelligence- (SI-) based optimization algorithms. Due to its robust and phase-divided structure, the ABC algorithm has been successfully applied to different types of optimization problems. However, some assumptions that are made with the purpose of reducing implementation difficulties about the sophisticated behaviours of employed, onlooker, and scout bees still require changes with the more literal procedures. In this study, the ABC algorithm and its well-known variants are powered by adding a new control mechanism in which the decision-making process of the employed bees managing transitions to the dance area is modeled. Experimental studies with different types of problems and analysis about the parallelization showed that the newly proposed approach significantly improved the qualities of the final solutions and convergence characteristics compared to the standard implementations of the ABC algorithms.

## 1. Introduction

Some species especially those living as swarms, societies, or colonies perform complicated behaviours in foraging, feeding, and surviving. When the behaviour of a specific individual is analyzed, it is seen that the mentioned behaviour of the individual is so simple compared to the complete task and it requires obeying only fundamental rules [1–3]. This property of a swarm to do with the combination of simple tasks of individuals in an order has gained the researchers' attention and various problem-solving techniques were proposed by utilizing bird, fish, wolf, and cat swarms or insect colonies including ants, termites, and bees [4–6].

The ABC algorithm that mimics the food foraging behaviours of real honey bee colonies is one of the most innovative and efficient contributions to the nature-inspired optimization algorithms [4, 5, 7]. At the beginning, the ABC algorithm was proposed for solving constrained and unconstrained numerical optimization problems, but it has been successfully applied to the combinatorial optimization problems in recent years. Although the ABC algorithm was capable of handling different types of problems with the standard working schema of it and proved its superiority compared with other evolutionary computational techniques, researchers from various fields presented new approaches for the ABC algorithm in order to further increase the convergence performance and qualities of the final solutions [4, 5].

When the studies about the ABC algorithm are investigated, it can be seen that all of these studies are roughly classified into three groups. In the first group of the studies, the ABC algorithm is tried to be strengthened by modifying the existing models that are related to the generation of initial and candidate food sources or solutions, calculation of selection probabilities, and so on. Tsai et al. integrated the Newtonian law of universal gravitation into the onlooker bee phase of the ABC algorithm and proposed interaction ABC, IABC, algorithm [8]. Zhu and Kwong proposed gbest-guided ABC, GABC, algorithm in which the food sources chosen by the onlooker bees are modified with the corresponding parameters of the global best food source in addition to the parameters of the randomly determined food source [9]. Akay and Karaboga used a probabilistic model for deciding whether a parameter is changed or not in the original search equation of the standard ABC algorithm and analyzed the performance of the proposed ABC algorithm on solving high-dimensional unconstrained optimization problems and constrained engineering problems [10]. Gao et al. introduced two ABC algorithm variants named ABC/best/1 and ABC/best/2 [11]. For both ABC/best/1 and ABC/best/2, the best food source is used as a guide when an employed or onlooker is sent from hive to find a candidate solution [11]. Xiang and An modified initial food source generation schema of the standard ABC algorithm with chaotic initialization and changed the roulette wheel selection mechanism with a reverse roulette wheel-based selection mechanism in order to sustain population diversity [12]. Luo et al. changed the probabilistic selection approach of the onlooker bee phase and proposed a new ABC algorithm variant called converge-onlookers ABC, COABC, algorithm [13]. Each onlooker bee in the OABC algorithm is sent to the vicinity of the global best food source to further increase the exploitation characteristics of the ABC algorithm [13]. Gao et al. changed the search equation, parameter utilization, and determination procedure of the basic ABC algorithm and named this modified variant as the OCABC algorithm [14]. For the OCABC algorithm, an employed or an onlooker produces candidate solution by using two randomly chosen food sources rather than using only one random food source [14]. In addition to this major change, they used the test and prediction abilities of the orthogonal learning approach to identify the related parameters being changed for generating new candidates [14]. In another study, Gao et al. brought advantages of different search mechanisms and multipopulation techniques together and introduced a new ABC algorithm called the ILABC algorithm [15]. In the ILABC algorithm, search equations used in the employed and onlooker bee phases are changed and the whole colony is divided into small subcolonies [15]. For each subcolony, the search equation of the employed bee phase is powered with the best solutions of the subcolony while the search equation of the onlooker bee phase is powered with the global best solution of the whole colony [15]. Qin et al. investigated the performance of the ABC algorithm when the number of employed and onlooker bees is changed with linear and nonlinear time-varying strategies [16]. Tran et al. remodeled the arithmetic crossover operator by controlling the candidate generation schema of the standard ABC algorithm and proposed enhanced ABC, for short EABC, algorithm [17].

The second group of studies mainly focuses on the hybridization of the ABC algorithm with other well-known population-based optimization techniques or local search approaches. Kang et al. combined the ABC algorithm with the Nelder–Mead simplex method for solving inverse analysis problems and introduced the Hybrid Simple ABC, HSABC, algorithm [18]. The HSABC algorithm was used as a parameter identification method for concrete dam-foundation systems and the results obtained by the HSABC algorithm were better than the results obtained by the standard ABC and real-coded genetic algorithm (RCGA) [18]. Ozturk and Karaboga hybridized the ABC algorithm with the Levenberg–Marquardt (LM) algorithm for finding optimal weight set of the neural networks [19]. In the proposed approach called the ABC-LM algorithm, the ABC algorithm is responsible for initial training of the neural network. The weight set determined by the ABC algorithm is used for further training by the LM algorithm [19]. Duan et al. proposed a new hybrid approach by utilizing the ABC algorithm and the Quantum Evolutionary algorithm (QEA) for solving continuous optimization problems [20]. In the proposed model, employed and onlooker bee phases of the ABC algorithm are modified with the candidate generation schema of the traditional QEA [20]. Zhao et al. integrated the ABC algorithm and genetic algorithm (GA) for a novel hybrid swarm intelligent approach called HSIGA [21]. The main idea of the HSIGA is based on information sharing between populations of two algorithms. Both GA and ABC algorithm start optimization with random individuals simultaneously, and selected individuals from two populations are crossovered at the end of the each cycle [21]. Finally, obtained offsprings are transferred to the population of GA. Kang et al. increased solving capabilities of the ABC algorithm with the Hookee Jeeves pattern search approach and introduced the Hookee Jeeves ABC, HJABC, algorithm [22]. Yan et al. used the crossover operator of the GA in the search equation of the ABC algorithm and names this new variant as the hybrid ABC, HABC, algorithm [23]. Mao et al. improved the performance of the standard ABC algorithm by combining it with the opposition-based learning, S-type subpopulation grouping and sensitivity-phenomenon option techniques [24].

ABC algorithm, like other population-based metaheuristics, is intrinsically suitable for running on distributed or shared memory-based parallel architectures. The studies in the third group are directly related with the parallelization of the ABC algorithm. Narasimhan parallelized the ABC algorithm in a manner that the whole colony is divided into equal-sized subcolonies and these subcolonies are assigned to the different processors [25]. At the end of each cycle, improved individuals are copied to the memory locations that are accessible to all the processors for increasing the utilization from the more qualified solutions [25]. Banharnsakun et al. used a distributed memory-based system for testing their parallel implementation of the ABC algorithm. In this parallelization approach, local best food sources between two randomly determined compute nodes are exchanged after completion of a predetermined number of cycles [26]. Parpinelli et al. investigated different parallelization approaches of the ABC algorithm including master-slave, multihive with migration and hybrid hierarchical [27]. Basturk and Akay first investigated the parallel ABC algorithm in which each bee works synchronously [28]. Secondly, they proposed a coarse-grained parallel model for the ABC algorithm and analyzed its performance on solving numerical optimization problems and training neural networks [29]. Karaboga and Aslan analyzed performance of the parallelized ABC algorithm by using newly proposed emigrant creation strategies [30]. They also investigated performance of the parallelized ABC algorithm on solving the motif discovery problem [31].

When the studies about the ABC algorithm are considered, it can be generalized that proposed modifications and hybridizations aim at changing source search and consumption characteristics of the employed, onlooker, and scout bees. However, intelligent behaviours seen in bee colonies are not limited with the search and consumption operations. Actually, search and consumption characteristics of the individuals in the colony are implicitly managed with the decisions about the transition to the dance area made by employed bees. In this study, a new model for the decision-making process of the employed bees called the transition control mechanism is introduced and integrated to the workflow of the standard ABC algorithm and its variants. The serial and parallelized implementations of the proposed ABC algorithm are tested on solving numerical benchmark problems with different number of parameters and wireless sensor deployment problem. The results from the experimental studies showed that modeling the decision-making process of the employed bees significantly improves convergence speed and quality of the final solutions of the ABC algorithm and has a decreasing effect on the execution time of the algorithm. The rest of the paper is organized as follows: fundamental steps of the ABC algorithm and bee phases are described in Section 2. The transition control mechanism and its adaptation to the ABC algorithm are summarized in Section 3.  Section 4 presents the results of the experimental studies. Finally, the conclusion is given in Section 5.

## 2. Artificial Bee Colony Algorithm

Some species living together like ants, fish, and birds in nature are capable of performing complex behaviours that are actually defined as a composition of simple operations carried out by individuals without requiring any centralized management or monitoring mechanism [1–3, 5]. These types of complex or intelligent behaviors have also been seen in the foraging habits of the real honey bee colonies. The intelligent foraging model in a bee colony consists of three fundamental components called food sources and employed and unemployed foragers. An employed forager is associated with a specific food source and responsible for carrying nectar to the hive. An employed forager also shares information about the food source such as nectar quality, distance, and direction with unemployed foragers [1–3, 5]. Unemployed foragers can be classified into two groups of bees. The first group of unemployed foragers is composed of onlooker bees [1–3, 5]. Onlooker bees wait on the hive and watch various dances performed by employed foragers before selecting a food source. Selection of a food source by an onlooker is not a random procedure directly. There is a relationship between choosability of a food source by onlookers and nectar quality of it. Another group of unemployed foragers is composed of scout bees. Scout bees wait on the hive like onlookers, but they fly from hive randomly or with the effect of internal or external triggers to find new food sources [1–3, 5]. By considering all of these specialized situations of a bee colony, Karaboga introduced a new swarm intelligence-based probabilistic optimization algorithm named ABC [1–3, 5]. In the ABC algorithm, positions of the food sources found and consumed by bees correspond to the possible solutions of the problem being optimized and nectar amount of a food source is directly related to the appropriateness of the solution [1–3, 5].

In the vast majority of the population-based optimization algorithms, a set of solutions is first created randomly and tried to be optimized until completion of a predetermined number of iterations, generations, or cycles [1–3, 5]. In the ABC algorithm, initialization of the optimization process is also devoted to the generation a set of solutions or food sources randomly by sending scout bees from the hive. The *jth* parameter of the *ith* food source within *SN* different food sources or solutions is determined randomly using equation (1) [1, 2, 32–34]. In equation (1), *x*_*ij*_ is the *j*th parameter of *D* different parameters in *i*th food source represented by *x*_*i*_, and *x*_*j*_^min^ and *x*_*j*_^max^ are lower and upper bounds of the *j*th parameter. Finally, rand (0, 1) is a random number between 0 and 1 [32, 33, 35]:

(1) $$\begin{array}{c} x_{ij}=x_{j}^{\min}+\text{rand}\left(0,1\right)\left(x_{j}^{\max}-x_{j}^{\min}\right),j=1,2,\ldots ,D,i=1,2,\ldots ,\text{SN}. \end{array}$$

After the discoveries of initial food sources by scout bees, these food sources are related with the employed bees for gathering nectars. In the ABC algorithm, each food source is assigned to only one employed bee and the location information of the food source is memorized by the associated employed bee. When an employed bee leaves from the hive, she tries to find another food source within the neighborhood of the memorized one. The search operation carried out by an employed bee for finding a new food source is modeled as in equation (2) for the ABC algorithm [1, 2, 32, 33, 35]:

(2) $$\begin{array}{c} v_{ij}=x_{ij}+\phi_{ij}\left(x_{ij}-x_{kj}\right), \end{array}$$

where *v*_*ij*_ is the *j*th parameter of the candidate food source *v*_*i*_ whose parameters are the same with the parameter values of the food source *x*_*i*_ except the *j*th one. *x*_*ij*_ and *x*_*kj*_ are the *j*th parameters of the *x*_*i*_ and *x*_*k*_ food sources, and *ϕ*_*ij*_ is a random number between −1 and 1 [1, 2, 32, 33, 35]. In addition to these, it should be noted that *j* and *k* are randomly determined indexes and *k* must be different than *i*. If the fit(*v*_*i*_) fitness value of the *v*_*i*_ food source calculated as in equation (3) for a minimization problem using the obj(*v*_*i*_) objective function value is higher than the fit(*x*_*i*_) fitness value of the *x*_*i*_ food source, the employed bee applies a greedy selection procedure between these food sources and a trial counter defined as trial_*i*_ is set to zero. Otherwise, *x*_*i*_ food source is still memorized by the employed bee and its trial counter is incremented by one to show that it is not improved in the current cycle [1, 2, 32, 33, 35]:

(3) $$\begin{array}{c} \text{fit}\left(v_{i}\right)=\left\{\begin{array}{cc} \frac{1}{\left(1+\text{obj}\left(v_{i}\right)\right)}, & \text{if obj}\left(v_{i}\right)>0\\ 1+\left|\text{obj}\left(v_{i}\right)\right|, & \text{if obj}\left(v_{i}\right)\leq 0 \end{array}\right\}. \end{array}$$

When all the employed bees turn back to the hive, they share the information about the visited food sources in their minds with the onlooker bees. Onlooker bees apply a selection procedure in which choosability chance of a food source increases with the fitness value of the same food source. The relationship between the fitness values of the sources and their choosabilities is modeled in the ABC algorithm by the assigned selection probabilities to all of these sources. The selection probability of the *x*_*i*_ food source defined as *p*(*x*_*i*_) is calculated by dividing the fit(*x*_*i*_) fitness value of the *x*_*i*_ food source with the sum of fitness values as given in equation (4) below. With the completion of the probabilistic food source selection, onlooker bees leave the hive and join the foraging as done by employed bees [1, 2, 32, 33, 35]:

(4) $$\begin{array}{c} p\left(x_{i}\right)=\frac{\text{fit}\left(x_{i}\right)}{\sum_{j}^{\text{SN}}\text{fit}\left(x_{j}\right)}. \end{array}$$

If the foraging characteristics of the employed and onlooker bees are investigated, it is clearly seen that utilization or exploitation of the existing food sources is more dominant than the exploration of new ones. However, exploration and exploitation processes should be managed in a balanced manner that satisfies acceptable convergence speed and solution diversity. This subtle balance between exploration and exploitation is maintained in the ABC algorithm by comparing the trial counters that are incremented or set to zero according to the improvements of the food sources with a specific control parameter named *limit*. If there is a food source for which its trial counter exceeds the *limit* parameter value at most, this food source is abandoned and the employed bee of the abandoned food source becomes the scout bee for finding a food source by using equation (1) [1, 2, 32, 33, 35]. Although there is no restriction on the values being assigned to the *limit* parameter, it can be calculated using the following equation by using the number of parameters and food sources [1, 2, 32, 33, 35]:

(5) $$\begin{array}{c} \left\lceil a\times \text{SN}\times D\right\rceil \text{ and }a\in {\mathbb{Q}}^{+}. \end{array}$$

By considering the detailed descriptions of the employed, onlooker, and scout bees, mathematical models for generating new and candidate food sources and fundamental steps of the ABC algorithm can be given as shown in Algorithm 1.

## 3. Transition Control Mechanism for Employed Bees of the ABC Algorithm

In the standard implementation of the ABC algorithm, an employed bee visits a new food source that is near to the location of the previously visited one. She applies a greedy selection between them and then turns back to the hive for sharing information about the memorized food source with the onlooker bees. However, in a real honey bee colony, information sharing procedures between employed and onlooker bees are not so straightforward and they are actually managed with nondeterministic behaviours of the employed bees. Even though all of the employed foragers turn back to the hive, some of them decide to visit dancing area for sharing information with the onlooker bees, while others leave the hive directly without informing any onlooker.

**Figure 1:**
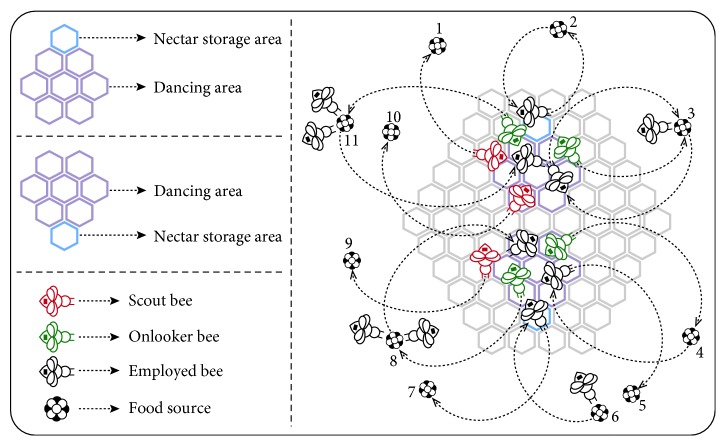
Different behaviours of the employed bees.

For understanding how the mentioned characteristics of employed bees change the bee density on the dance area, Figure 1 can be further investigated. As seen from Figure 1, the employed bees that visit the sources numbered 3, 4, 8, and 11 share the information about these food sources with the onlooker bees on the dance area. However, the employed bees that visit the sources numbered 2 and 6 do not share the information about these food sources with onlooker bees even though they turn back to the hive near or at the same time when compared to the other employed bees. Although the reason lying behind this type of decision made by an employed bee cannot be known directly, it has an important effect on the source selection procedure of the onlooker bees. If a group of employed bees leave the hive without introducing the visited food sources while the remaining employed bees do, the entire set of food sources that will be utilized by the onlooker bees is reduced to a specified set of food sources. In the first sense, the reduction of the entire set of food sources to a specified set of food sources can be thought as a drawback for the selection process of the onlooker bees, but the specialized set of solutions, in which only food sources introduced by the employed bees on the dance area can be found, contains more eligible sources than the extracted ones. In this situation, the chance of an onlooker to do with the utilization from more suitable solution or solutions is increased significantly.

If the decision-making mechanism of the employed bees that manages whether she visits the dance area or not is modeled and integrated to the standard implementation of the ABC algorithm, the search characteristics and complex behaviours of employed bees are reflected more robustly and the waste of computing resources with weak food sources is minimized. The proposed approach in this study that is also called the transition control mechanism for short tl directly focuses on the modeling of the given properties of the employed bees. In the tl mechanism, when the employed bee phase is completed, the decision whether an employed bee goes through the dance area or not is made by comparing the fitness value of the memorized food source with the average fitness value of the entire set of food sources. If the fitness value of the food source is less than the average fitness value, the employed bee associated with that food source is not directed to the dance area.

The average fitness value obtained by dividing the sum of the fitness values of the food sources to the number of employed bees can be adjusted by multiplying it with a constant value ranging between 0 and 1. This constant value in tl is named as the transition factor (tf). While the size of the reduced set of food sources increases with the lower values of the tf parameter, qualities of the transitioned sources can be enhanced with the higher values of it. In tl, calculation of the average fitness value and its adjustment with the tf parameter can be made by using equation (6). In equation (6), *tlDecision*_*i*_ shows the transition decision of the employed bees related with the *x*_*i*_ food source. If *tlDecision*_*i*_ value is equal to 1, the employed bee related with the *x*_*i*_ food source moves towards dance area. Otherwise, she flies from hive without informing any onlooker:

(6) $$\begin{array}{c} tlDecision_{i}=\left\{\begin{array}{cc} 1, & \text{if fit}\left(x_{i}\right)\geq \text{tf}\times \frac{\left(\sum_{j}^{\text{SN}}{\text{fit}}_{j}\right)}{\text{SN}}\\ 0, & \text{if fit}\left(x_{i}\right)<\text{tf}\times \frac{\left(\sum_{j}^{\text{SN}}{\text{fit}}_{j}\right)}{\text{SN}} \end{array}\right\}. \end{array}$$

When tl is applied to the ABC algorithm, from now on, the ABC algorithm powered by the tl is called the tlABC algorithm, it should be noticed that the reduced set of food sources for the onlooker bee phase can contain only one solution. For the candidate generation model of the ABC algorithm, a food source except the used one is needed at least. By considering the limitation stemmed from the candidate generation schema, the tlABC algorithm tries to improve the transitioned food source with a randomly determined food source when the number of food sources in the reduced set after transition control is equal to one. For detailed description of the transition control approach and its usage with the ABC algorithm, fundamental steps given in Algorithm 2 can be analyzed.

## 4. Experimental Studies

For analyzing the effect of the newly proposed mechanism called transition control on the solution quality, convergence characteristics, and finally execution time of the ABC algorithm, experimental studies carried out were divided into three different groups. Standard implementation of the ABC algorithm and its transition-controlled variant, tlABC, were compared over a set of well-known numerical benchmark problems in the first group of the experimental studies. Also, possible contributions of the tl mechanism were analyzed over the well-known variants of the ABC algorithm including GABC [9], ABC/best/1 [11], ABC/best/2 [11], CABC [14], and EABC [17] in the first group of the studies.

The ABC algorithm can be parallelized for working on distributed or shared memory-based architectures by applying a limited set of modifications. By considering this advantage, parallelization of the ABC algorithm powered by new approach should also be investigated. In the second group of the experimental studies, ABC and tlABC algorithms were parallelized by dividing the whole colony into subcolonies running simultaneously for utilizing from the computational power of a multicore system and tested for solving numerical problems used in the previous group of the experiments. In the final group of the experimental studies, performances of the standard ABC and tlABC algorithms were compared through solving an NP-hard problem called wireless sensor deployment. In the wireless sensor deployment problem, positions of the wireless sensor nodes are determined in a manner that the coverage of the network will be maximized as much as possible. Results of the experimental studies in this group also helped analyze transition control mechanism for a different type of problem.

### 4.1. Solving Numerical Optimization Problems with Transition-Controlled ABC Algorithms

In order to analyze the effect of the transition controlling on the search and convergence characteristics of the standard ABC algorithm, nine different benchmark functions are chosen [10]. *f*_1_, sphere function, is a continuous benchmark function and has one global minimum. *f*_2_, step function, is similar to the *f*_1_ function except that it is not continuous. *f*_3_, Schwefel function, contains multiple peaks and valleys. *f*_4_, Rosenbrock's valley function, is one of the most commonly used benchmark problems to analyze the convergence characteristics of the optimization algorithms. For this function, the global minimum is located at the end of a long, narrow, and flat valley. *f*_5_, Dixon-Price function, is another unimodal and continuous function. *f*_6_, Rastrigin function, is based on the sphere function but it has many local minimas distributed to the search space. *f*_7_, Griewank function, is the multimodal benchmark problem and local minimas change with the dimensionality. *f*_8_, Ackley function, contains an exponential term that generates many local minimas. Finally, *f*_9_, penalized, is a compute-expensive problem and requires calculations of a fundamental trigonometric function. For all of the benchmark problems except *f*_3_, the global minimum value is equal to 0. Global minimum of the *f*_3_ function is related with the number of parameters. Given that the number of parameters is equal to *D*, global minimum value of *f*_3_ can be found as −*D* × 418.9829. Lower and upper bounds of the parameters and formulations of the functions are given in Table 1.

In all experiments, the size of the colony is determined as 100 and the whole colony is equally divided between employed and onlooker bees [10]. All of the nine benchmark functions are tested with dimensions 10, 100, and 500. Maximum fitness evaluation, MFE, is taken equal to 100, 000 and 1, 000, 000, respectively, for understanding short- and long-term response of the proposed model. For a detailed investigation how the transition factor changes the results of the tlABC algorithm, four different values of tf including 0.25, 0.50, 0.75, and 1.00 are used. The *limit* parameter value is calculated by considering the number of parameters (*D*) and number of food sources (SN) as SN × *D* [10]. Each of the experiments is repeated 30 times with different random seeds by using a system that is equipped with a four-core Intel® i5 750 processor running 2.66 GHz with 4 GB of RAM. The mean best values produced by the algorithms and standard deviations over 30 runs are given in Tables 2–7.

From the results given in Table 2, it can easily seen that standard ABC and tlABC algorithms are capable of finding the global optimum values for the *f*_2_, *f*_3_, *f*_6_, and *f*_7_ functions. The mean best objective function values of *f*_8_ and *f*_9_ are all same for ABC and tlABC with tf=0.25, tf=0.50, tf=0.75, and tf=1.00, while the tlABC algorithm produces a better mean best objective function values for *f*_1_, *f*_4_, and *f*_5_. When difficulties of the problems are changed by increasing the number of parameters, effectiveness of the tl model becomes more apparent. The results in Table 3 prove the obtained improvements with the tlABC algorithm. For the *f*_3_, *f*_4_, *f*_5_, *f*_6_, *f*_7_, *f*_8_, and *f*_9_ functions, the tlABC algorithm produces better results compared to the standard ABC algorithm. While the mean best objective function values of the *f*_2_ function are all the same for ABC and tlABC algorithms, the standard ABC algorithm is better than the tlABC algorithm for only *f*_1_ function. By considering mean best objective function values given in Table 4, it can be generalized that the value of the tf parameter should be chosen very close or equal to 1.00. For all of the nine benchmark functions with 500 parameters, the tlABC algorithm produces better results than the ABC algorithm by setting the tf parameter value to 1.0.

The results in Tables 5–7 provide important information about the solving capabilities of the tlABC algorithm and effect of the tl model on high number of fitness evaluations. When Table 5 that shows the results on optimizing 10-dimensional problems until completion of 1, 000, 000 fitness evaluations is analyzed, it is seen that there is no difference between ABC and tlABC algorithms in terms of mean best objective function values for *f*_1_, *f*_2_, *f*_3_, *f*_6_, *f*_7_, and *f*_9_ functions. While the only 10-dimensional function for which ABC outperforms tlABC algorithm is *f*_5_, tlABC with tf=0.50 and tlABC with tf=1.00 outperforms the standard ABC algorithm and other tlABC variants for *f*_4_ and *f*_8_, respectively. Although the number of parameters is increased 10 times compared to the previous experimental case, there are only three functions for which ABC and tlABC algorithms produce different mean best objective function values from each other. While the tlABC algorithm obtains better results than the ABC algorithm for *f*_4_ and *f*_8_ functions, the ABC algorithm outperforms the tlABC algorithm for the *f*_1_ function. Finally, when Table 7 that shows the results on optimizing 500-dimensional problems until completion of 1, 000, 000 evaluations is considered, it is seen that the tlABC algorithm produces better results than the ABC algorithm on eight of nine benchmark functions. For *f*_3_, *f*_6_, and *f*_8_ functions, the tlABC with tf=1.00 outperforms the ABC algorithm and tlABC algorithm with other tf values, while tlABC with tf=0.75 produces better results than the standard and other tlABC algorithms used in comparison for *f*_1_ and *f*_3_ functions. In addition, tlABC with tf=0.25 is best among other variants for *f*_4_ and *f*_5_ functions while tlABC with tf=0.50 outperforms the standard ABC and other tlABC algorithms with different tf values for the *f*_7_ function.

Although mean best values produced by the tlABC algorithm with different values of tf are generally better than the standard implementation of the same algorithm, mentioned efficiency of the tlABC algorithm should also be proven with statistical methods. So, in order to determine that the difference between algorithms is whether significant or not, a nonparametric test called Wilcoxon signed-rank test is used with the significance level of 0.05 (*p* ≤ 0.05). The results of the test are given in Tables 8 and 9 for the most challenging situation that contains 500-dimensional functions. When the results given in Table 9 for 100, 000 function evaluations are considered, it is concluded that the difference between ABC and tlABC with tf=1.00 is significant in favor of the tlABC algorithm for all of the nine benchmark functions. While the difference between ABC and tlABC is also significant in favor of the tlABC algorithm with tf=0.75 for *f*_3_ and *f*_4_ functions, the difference between these algorithms is not meaningful for other functions.

The results of the Wilcoxon signed-rank test obtained by analyzing best objective function values found after completion of 1, 000, 000 fitness evaluations for 500-dimensional functions in Table 8 also showed that the differences between ABC and tlABC algorithms are significant in favor of tlABC algorithms for *f*_1_, *f*_7_, and *f*_9_ functions. While the tlABC algorithm with tf=1.00 is still statistically significant compared to the ABC algorithm for *f*_3_, *f*_6_, and *f*_8_, the only function for which the ABC algorithm is statistically significant than tlABC with tf=1.00 is *f*_4_. The difference between ABC and tlABC with tf=0.75 algorithm is not meaningful for *f*_2_, *f*_4_, *f*_5_, *f*_6_, and *f*_8_ functions. Finally, the tlABC with tf=0.75 is statistically significant than the ABC algorithm for the *f*_3_ function in addition to the *f*_1_, *f*_7_, and *f*_9_ functions.

**Figure 2:**
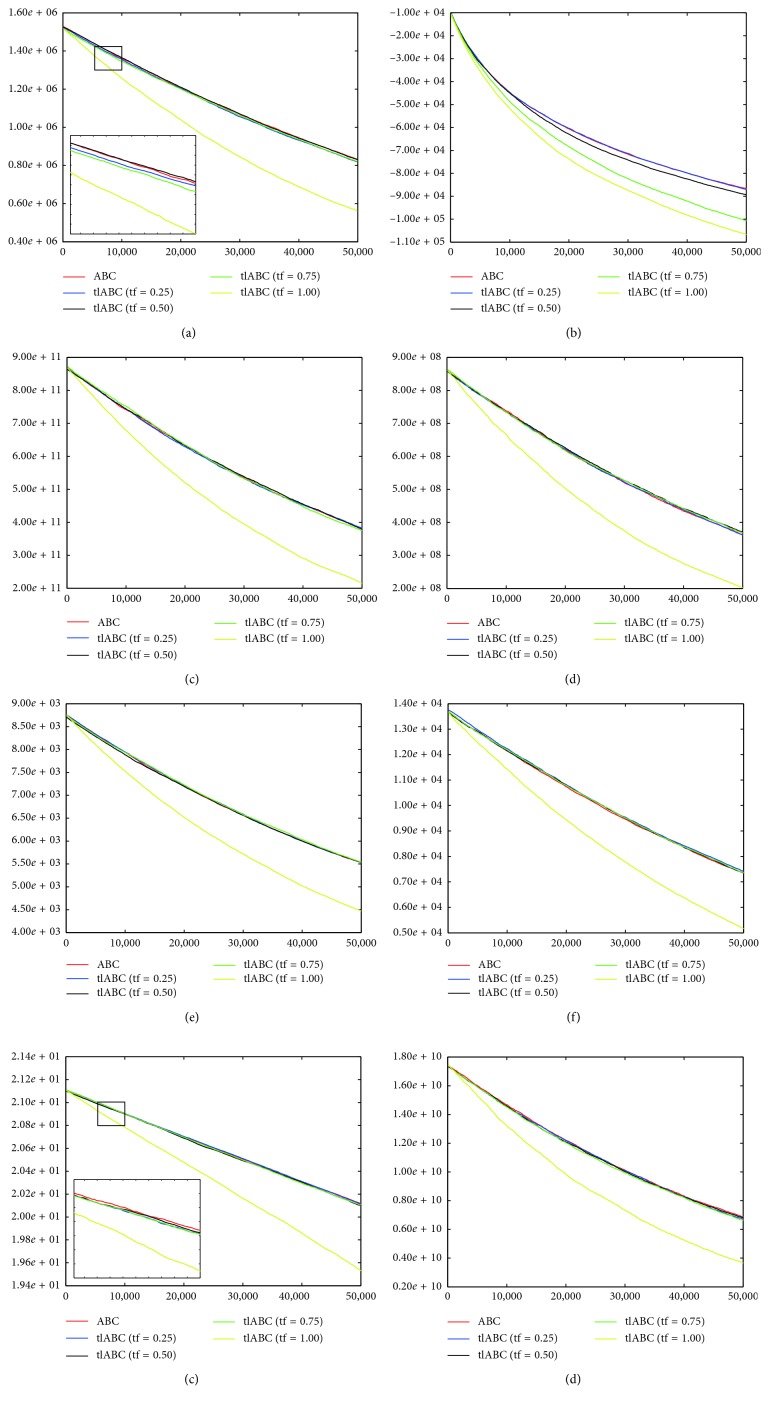
Convergence graphics of 500-dimensional *f*_1_ (a), *f*_3_ (b), *f*_4_ (c), *f*_5_ (d), *f*_6_ (e), *f*_7_ (f), *f*_8_ (g), and *f*_9_ (h) functions for ABC and tlABC algorithms.

By controlling employed bee transitions with newly proposed model, the chance of utilizing more qualified solution or solutions in the onlooker bee phase increased compared to the standard workflow of the algorithm. For analyzing the effect of the tl model on the convergence characteristics of the ABC algorithm, the graphics given in Figure 2 can be examined for 500-dimensional *f*_1_, *f*_3_, *f*_4_, *f*_5_, *f*_6_, *f*_7_, *f*_8_, and *f*_9_ functions. For all of the convergence graphics in Figure 2, the *x* and *y* coordinates correspond to the fitness evaluations and mean best objective function values, respectively. From the graphics, it can be generalized that the tf parameters less than 1.00 cannot produce a remarkable change on the convergence performance of the tlABC algorithm compared to the standard ABC implementation at the first 50, 000, fitness evaluations. However, when the tf parameter value is set to 1.00, convergence speed of the tlABC algorithm increases significantly compared to the standard ABC and other tlABC algorithms for all of the illustrated benchmark functions.

The positive contribution of the tl mechanism on the convergence speed can also be proven by counting the consumed fitness evaluations until reaching the predetermined threshold objective function value [14]. With this purpose, the number of fitness evaluations consumed to reach the determined threshold is recorded for each 30 independent runs. If the required number of fitness evaluations is less than or equal to 1, 000, 000 the algorithm is accepted as successful for the mentioned run. The threshold values of the 500-dimensional *f*_1_, *f*_2_, and *f*_7_ functions are determined as 1.0*e* − 06, while the threshold values of the 500-dimensional *f*_8_ and *f*_9_ functions are set to 1.0*e* − 02. For the 500-dimensional *f*_4_ function, −1.0*e*+5 is chosen as the threshold value. Finally, the threshold values of the 500-dimensional *f*_4_, *f*_5_, and *f*_6_ functions are determined as 1.0*e*+04, 1.0*e*+03, and 1.0*e*+02, respectively. In Table 10, success rates (Sr) of the algorithms and mean fitness evaluations (Me) of the runs found as successful are summarized. When Table 10 is analyzed, it is clearly seen that there is a certain correspondence between the convergence curves in Figure 2 (Sr and Me values). For all of the nine benchmark functions, the tl mechanism-based ABC algorithms require less fitness evaluations compared to the standard ABC algorithm.

As mentioned before, the tl mechanism controls the transition decisions of the employed bees to the dance area. Because of the reason that transition decisions are the common part of the employed bee phases of all ABC-based optimization techniques, it can be successfully used with the variants of the ABC algorithm in addition to the standard ABC algorithm. For investigating the possible contributions of the tl mechanism on the solving capabilities of the ABC variants, it is integrated to the workflows of the five well-known ABC algorithms including GABC [9], ABC/best/1 [11], ABC/best/2 [11], CABC [14], and EABC [17]. GABC, ABC/best/1, ABC/best/2, CABC, EABC, and their tl-based variants tlGABC, tlABC/best/1, tlABC/best/2, tlCABC, and tlEABC algorithms are tested with the benchmark functions given in Table 1. In all experiments, the size of the colony is determined as 100 and number of parameters is set to 500, while the maximum fitness evaluation is taken equal to 100, 000. The value of the tf parameter is taken equal to 1.0 and the *limit* parameter value is calculated as (SN × *D*). Each of the experiments is repeated 30 times with different random seeds on the system previously introduced. The mean best values produced by the algorithms and standard deviations over 30 runs are given in Table 11.

From the results given in Table 11, it is clearly seen that all of the tl-based ABC variants are capable of producing better mean best objective function values compared to their standard implementations. Although the tl mechanism improves the solving capabilities of the tested ABC variants, its effect changes according to the problem being solved and existing properties of the algorithms. While the improving effect of the tl mechanism on the performances of the ABC variants are similar for *f*_1_, *f*_2_, *f*_6_, *f*_7_, and *f*_8_ functions, the tl mechanism significantly increases the solving capabilities of the ABC/best/1 and CABC algorithms for the *f*_3_ function; CABC and EABC algorithms for *f*_4_ and *f*_5_ functions; and ABC/best/1, CABC, and EABC algorithms for the *f*_9_ function. In order to investigate that the tl mechanism is also capable of generating a statistical difference between GABC, ABC/best/1, ABC/best/2, CABC, EABC, and their tl-based variants, the results of the Wilcoxon signed-rank test with the significance level 0.05 (*p* ≤ 0.05) given in Table 12 can also be analyzed. The results in Table 12 showed that the superiority of the mean best objective function values obtained by the tl model-based GABC, ABC/best/1, ABC/best/2, CABC, and EABC algorithms is statistically proven against the mean best objective function values obtained by the standard implementations of the same algorithm for all of the nine benchmark functions.

**Figure 3:**
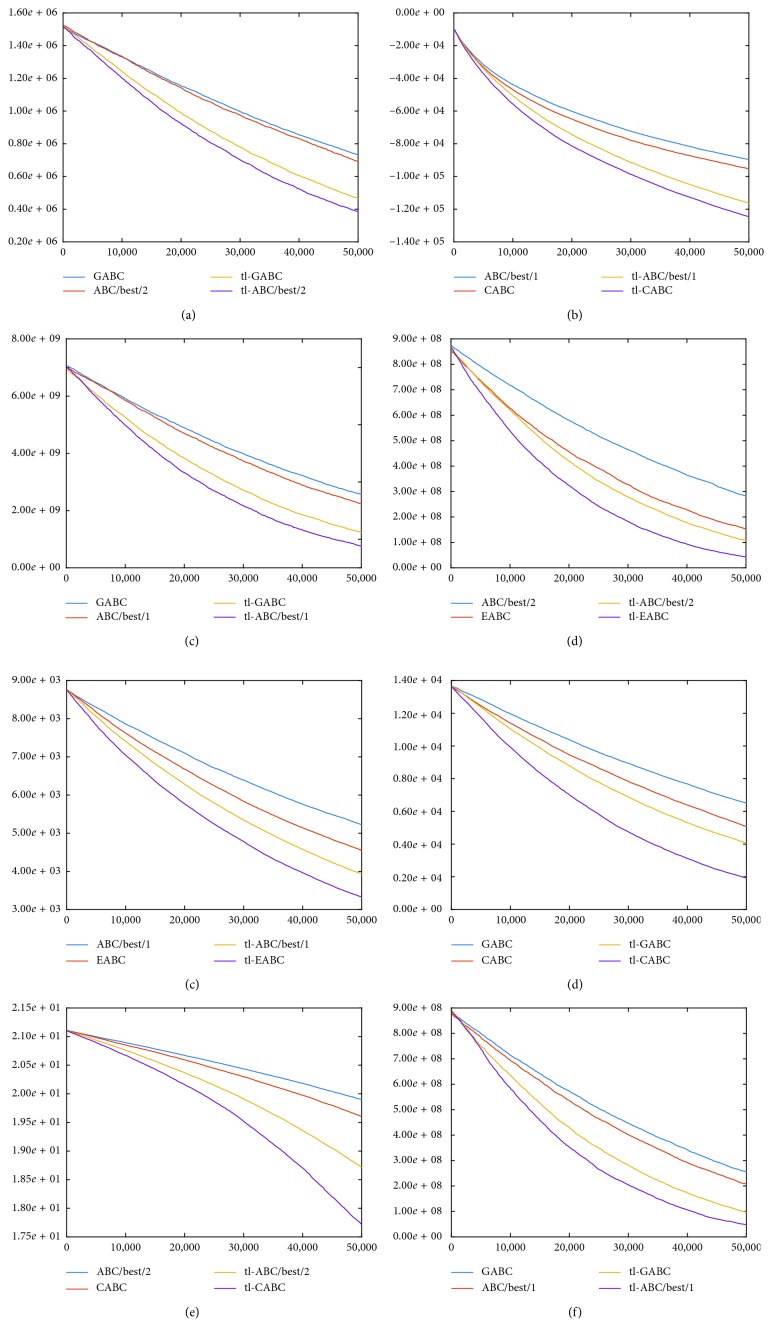
Convergence graphics of 500-dimensional *f*_1_ (a), *f*_3_ (b), *f*_4_ (c), *f*_5_ (d), *f*_6_ (e), *f*_7_ (f), *f*_8_ (g), and *f*_9_ (h) functions for ABC variants and their tl-based implementations.

In order to investigate how the tl mechanism changes the convergence characteristics of the GABC, ABC/best/1, ABC/best/2, CABC, and EABC algorithms, the graphics in Figure 3 should be controlled. From the graphics given in Figure 3, it can be said that the tl mechanism also improves the convergence curves of the tested ABC algorithms compared to their fundamental implementations. Although GABC, ABC/best/1, ABC/best/2, CABC, and EABC algorithms modify several states of the standard ABC algorithm and have different exploration and exploitation characteristics, the tl mechanism improves their solving capabilities all of them in terms of qualities of the final solutions and convergence speeds.

### 4.2. Solving Numerical Optimization Problems with Parallel Transition-Controlled ABC Algorithm

Driven by the increasing number of cores in a single processing unit and decreasing the setup costs of distributed memory-based systems, parallelization of the well-known techniques or their improved variants becomes a necessity. In order to analyze the possible contributions of the tl mechanism on the parallel implementations, a set of experimental studies is also carried out. For parallelization of the tlABC and standard ABC algorithms, one of the most commonly used approach that is based on dividing the whole colony into equally sized subcolonies and evaluating them on a unique compute node or core is utilized. However, this parallelization approach can deteriorate the required population diversity and lead to early convergence to local minimas. In order to address these drawbacks stemmed from the parallelization, subcolonies, or subpopulations, send-receive individuals based on the chosen neighborhood topologies. For the experiments, ring migration topology in which the *ith* subcolony sends its emigrant to the ((*i*+1)%*N*)th subcolony where *N* shows the number of subcolonies, cores, or computing nodes is used. The best food source of the current subcolony is chosen as an emigrant, and this emigrant source is exchanged with the worst food source in the neighbor subcolony.

For tlABC with tf=1.00 and ABC algorithms, the colony size is equal to 200 and maximum evaluation number is chosen as 200, 000 and 500, 000 for solving the 500-dimensional functions given in Table 1. The number of subcolonies for parallel ABC, p-ABC, algorithm and parallel tlABC, p-tlABC, algorithm is determined as 4 by considering the four-core processor of the running environment. ABC, p-ABC, tlABC, and p-tlABC algorithms are written in C programming language, and built-in functions of the *pthreads* library are used for parallelization and require synchronization between subcolonies. After completing 25 successive employed-onlooker-scout bee phase triple, a subcolony sends its current best solution to the neighbor subcolony and the received emigrant is replaced with the local worst solution. Each of the experiments is repeated 30 times with different random seeds and mean best objective function values and standard deviations are recorded and presented in Tables 13 and 14.

When the results given in Tables 13 and 14 are investigated, the superiority of the tlABC with tf=1.00 algorithm can be easily seen. For all of the nine benchmark functions, the tlABC algorithm produced better results compared to the ABC, p-ABC, and p-tlABC algorithms with the completion of 200, 000 or 500, 000 fitness evaluations. However, it should be noted that the p-tlABC algorithm are the second best for all of the benchmark functions considering the results in Table 13. When the number of fitness evaluations is increased 2.5 times compared to the first test scenario, the p-tlABC algorithm loses its advantages over the standard serial ABC algorithm especially for the *f*_4_, *f*_5_, and *f*_9_ functions, while it produces better results for *f*_2_, *f*_3_, *f*_6_, and *f*_8_ functions than the serial ABC algorithm. Finally, from the results in Table 14, it is seen that the p-tlABC algorithm still preserves its superiority compared to the p-ABC algorithm for all of the used benchmark functions.

Although the mean best objective function values of the p-tlABC algorithm showed that the tl mechanism protects its contribution against serial and parallel implementations of the standard ABC algorithm, these improvements should also be statistically validated. In Tables 15 and 16, the p-tlABC algorithm is compared with the ABC, p-ABC, and tlABC algorithms by using the Wilcoxon signed-rank test with the significance level of 0.05 (*p* ≤ 0.05). From the test results, the difference between p-ABC and p-tlABC algorithms is meaningful and in favor of the p-tlABC algorithm for all of the benchmark functions used in the experiments. When Table 15 is considered, the p-tlABC algorithm proves its superiority over ABC algorithm also with the statistically significant differences. With the increased total number of evaluations, the difference between ABC and p-tlABC algorithms starts to be negligible especially for the *f*_1_ and *f*_2_ functions. The difference between ABC and p-tlABC algorithms is significant in favor of the p-tlABC algorithm for *f*_3_, *f*_6_, and *f*_8_ functions, while the difference between these algorithms is significant in favor of the ABC algorithm for *f*_4_, *f*_5_, *f*_7_, and *f*_9_ functions when the termination criterion is determined as the 500, 000 fitness evaluations. The final extraction from the statistical test results is that the difference between serial and parallel implementation of the tlABC algorithms is significant in favor of the serial tlABC algorithm for all of the functions.

One of the important contributions by parallelizing an algorithm is to decrease total execution times as possible. In order to analyze how the execution times of the parallelized ABC algorithms change compared to the their serial counterparts, total elapsed times required for the completion of the determined number of fitness evaluations are recorded in terms of seconds for all of the 30 different runs and then average execution times and related standard deviations are given in Tables 17 and 18. When the results given in the tables are investigated, it should be first noticed that average execution times of the tlABC algorithm is less than the average execution times of the standard ABC algorithm. Although common parameters including colony size and maximum number of fitness evaluations and search equations used in employed and onlooker bee phases are the same for ABC and tlABC algorithms, the difference between average execution times of the ABC and tlABC algorithms in favor of the tlABC algorithm is originated from the quasi-deterministic structure of the tl mechanism. The onlooker bee phase of the standard ABC algorithm is nearly completed when all of the onlookers select sources. However, selection of a food source by an onlooker is a probabilistic process and the time required for selection cannot be estimated easily. In addition to this, the selection procedure is performed over all food sources introduced by the employed bees. If the majority of the food sources do not have high fitness values, only selection of the food sources can require more computations compared to the candidate generation and fitness calculation. But, in the tl mechanism, there is only one employed bee transitioned to the dance area or transitioned employed bees have more selection probabilities compared to the extracted ones.

Another comparison between serial and parallel implementations of the ABC algorithms can be made over commonly used metrics called speedup and efficiency. The speedup value can be defined as a ratio between execution times of the serial and parallel implementations of the considered algorithm, and its maximum value is equal to the number of cores or computing units. The efficiency value can be found by dividing speedup metric to the number of cores or computing units, and its maximum value can be 1.0. When the speedup and efficiency values given in Tables 19 and 20 for 200, 000 and 500, 000 evaluations are analyzed, there is only one function for which the efficiency value is less than 0.50.

**Figure 4:**
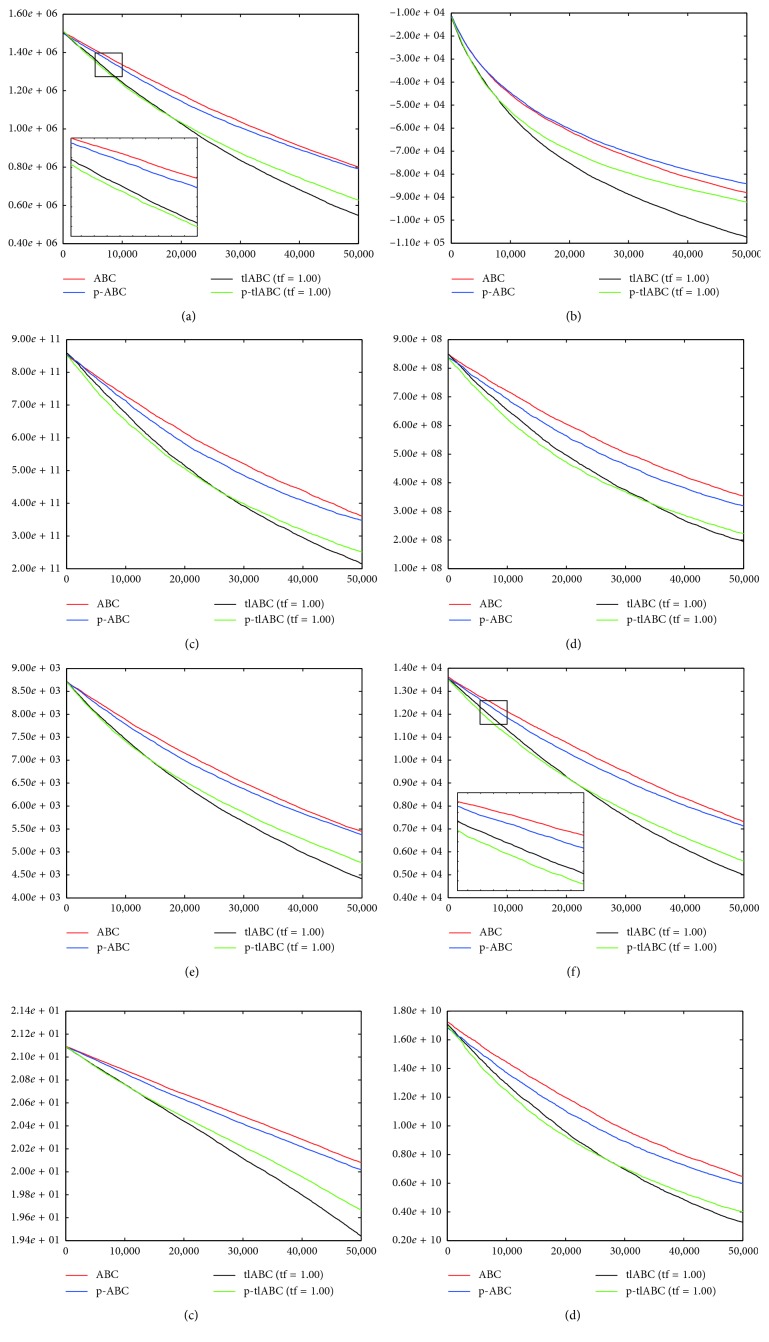
Convergence graphics of 500-dimensional *f*_1_ (a), *f*_3_ (b), *f*_4_ (c), *f*_5_ (d), *f*_6_ (e), *f*_7_ (f), *f*_8_ (g), and *f*_9_ (h) functions for serial and parallel ABC and tlABC algorithms.

A final comparison between serial and parallel implementations of ABC and tlABC algorithms is made over the convergence curves of them given in Figure 4. By considering the graphics represented in Figure 4, it can be generalized that serial tlABC with tf=1.00 has the most robust convergence characteristics among the ABC, p-ABC, and p-tlABC algorithms. However, it should be noticed that the p-tlABC algorithm obtains a few quicker convergence especially for the *f*_4_, *f*_5_, *f*_7_, and *f*_9_ functions within the first half of the 50,000 fitness evaluations. In the p-tlABC algorithm, each subcolony gets chance to utilize more qualified solutions with the tl model. Although increased number of solutions being utilized in the onlooker bee phase compared to the single colony configuration accelerates the convergence speed of the p-tlABC algorithm, the diversity in a subcolony is not enough to change the transitioned food sources for the subsequent evaluations.

### 4.3. Solving Sensor Deployment Problem with Transition-Controlled ABC Algorithm

A wireless sensor network contains hundreds or sometimes thousands of stationary or mobile sensor nodes. Each sensor node is usually capable of sending-receiving information obtained from the environment being monitored or targets being tracked. Although sensor nodes are versatile devices, they have limited sensing, computing, and storage capabilities and required power for detecting, and communication is maintained with a small battery. By considering all of these restrictions, the settlement of a wireless sensor network should be made in a manner that the coverage area of the sensor network or its lifetime is maximized. The area that is successfully covered by the sensor network or its utilization time is directly related with the exact positions of the sensor nodes.

Assume that the area being tracked is equally divided into small grids or rectangular subareas; *P* is a corner point of a grid, and this point is located at the (*x*, *y*) coordinate. The decision whether the point *P* is covered by the sensor *s*_*i*_ positioned at (*x*_*i*_, *y*_*i*_) can be made by comparing the Euclidean distance between sensor *s*_*i*_ and point *P*, dis(*P*, *s*_*i*_), with the detection range, *r*, of the same sensor. If the Euclidean distance dis(*P*, *s*_*i*_) is less than the *r* value, it can be said that the point *P* is covered by the sensor *s*_*i*_. Given that *c*_*i*_ shows the set of points that are successfully covered by the sensor *s*_*i*_ and the size of the area in which *S* different sensor nodes are deployed is *A*, the coverage ratio (CR) of the wireless sensor network can be calculated as shown in the following equation:

(7) $$\begin{array}{c} \text{CR}=\frac{\cup c_{i}}{A},\;i\;\epsilon \;S. \end{array}$$

When the ABC algorithm is used to find optimal or near optimal sensor deployment, a possible deployment is related with a food source and coverage rate of the deployment is matched with the objective function of the solution. In addition to these, it should be noted that a solution or a food source for the deployment problem consisting of *S* wireless sensors is represented with a vector of 2 × *S* elements for two-dimensional terrains and 3 × *S* elements for three-dimensional terrains.

The solving capabilities of the serial and parallel implementations of the ABC and tlABC algorithms on the deployment problem are tested for locating 100 mobile sensors in an area of 10, 000(100 × 100)*m*^2^. The colony size is 20, and the value of the *limit* parameters is equal to 100. For parallel implementations of ABC and tlABC algorithms, the whole colony is divided into two equal subcolonies and only two cores of the previously mentioned running environment are used. The ring migration topology is chosen as a neighborhood topology. The migration is carried out after completing 25 successive employed-onlooker-scout bee phase triples. Finally, the tf parameter of the tlABC algorithm is set to 1.00. Each of the experiments is repeated 20 times with different random seeds until completion of 2, 000 and 10, 000 evaluations. The best, mean, and worst coverage values and standard deviations are recorded and then summarized at Table 21. The results given in Table 21 also represent that the tlABC algorithm with tf=1.00 produces better deployments than other serial and parallel variants of the ABC algorithm considering the best, mean, and worst coverage values for both 2, 000 and 10, 000 fitness evaluations. Although the differences between tlABC algorithm and others are relatively small, the complexity of the deployment problem causes a long stagnation period that cannot easily be skipped with the current workflow of the ABC algorithm. However, the tlABC algorithm enters that period with the more robust solutions and slightly improves its current best solution until reaching the defined termination criteria.

Average execution times of the ABC-based deployment techniques in terms of seconds are presented in Table 22. Because of the colony size is chosen quite small compared to the previous experimental cases, average execution times of standard and tl model-based ABC algorithms are relatively close to each other. However, when the differences between fitness values of the colonies are apparent as in the case for which the termination criteria is equal to 2, 000 fitness evaluations, the tl model slightly reduces the average execution times with its quasi-deterministic selection procedure.

**Figure 5:**
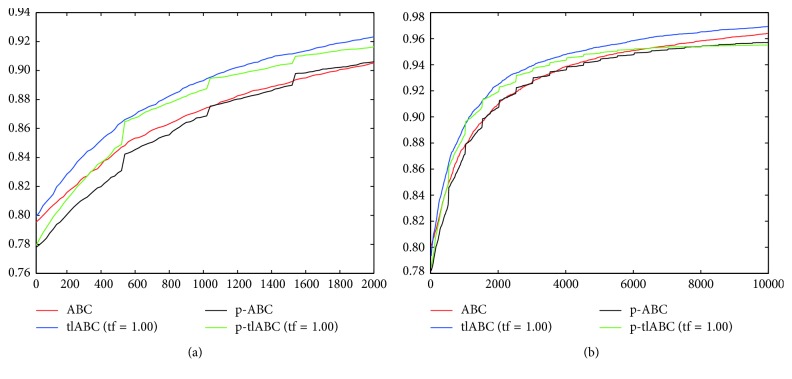
Convergence curves of ABC algorithms for 2, 000 (a) and 10, 000 (b) evaluations.

Finally, ABC and tlABC algorithms are compared on the basis of their convergence characteristics. As seen from the graphical representation given in Figure 5, the best convergence performance belongs to the serial tlABC algorithm. In addition to this, the effect of the information sharing on the convergence speed of the parallelized algorithms can be easily seen. With the distributions of the emigrant food sources between subcolonies, mean best coverage values of both p-ABC and p-tlABC algorithms increase gradually.

## 5. Conclusion

In this study, a new approach that models the decision-making mechanism of the employed foragers is introduced. The model called the transition control mechanism, for short tl, is integrated to the standard serial and parallel implementations of the ABC algorithm in addition to the well-known ABC variants. The possible contributions of the tl mechanism on the performance of the ABC algorithm are analyzed by solving numerical and combinatorial optimization problems. Experimental studies showed that the transition control mechanism significantly improved the searching capabilities of the standard and modified ABC algorithms. For the standard ABC algorithm, all of the employed bees are directed to the dance area in order to inform onlooker bees waiting on the hive. However, in a real honey bee colony, some of the employed bees leave the hive without visiting the dance area while some of them stay in the hive to interact with the onlooker bees. By adding the mentioned characteristics of the employed bees with the tl mechanism to the existing structure of the ABC algorithms, relationship between different group of bees are simulated more conveniently. In future, more efficient transition control mechanisms that determine whether an employed bee is directed to the dance area or not can be further studied.

## Figures and Tables

**Algorithm 1:**
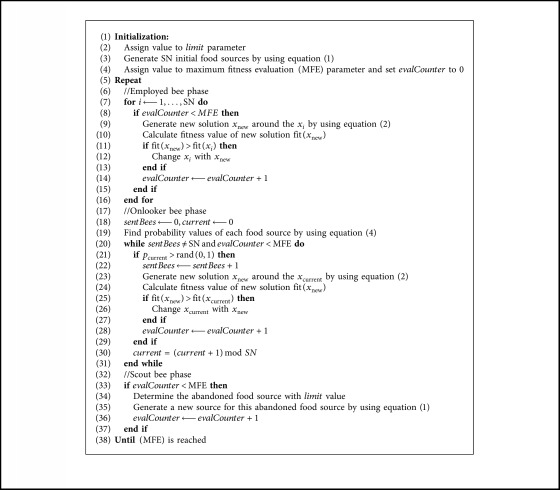
Fundamental steps of the ABC algorithm.

**Algorithm 2:**
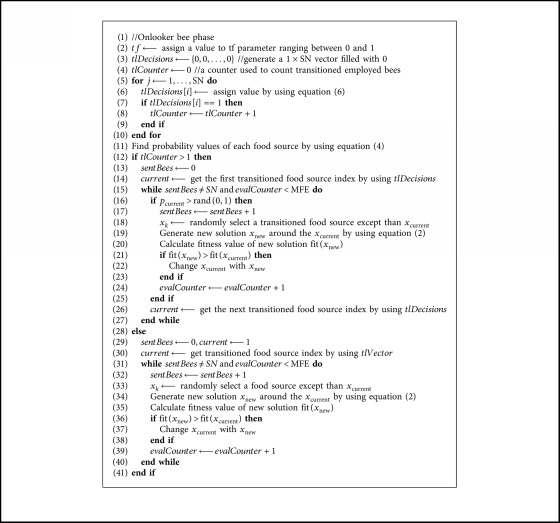
Onlooker bee phase with the tl mechanism.

**Table 1:** Benchmark functions used in experiments.

Function	Range	Formulation
Sphere	[−100, 100]	$f_{1}\left(\vec{x}\right)=\sum_{i=1}^{D}\left(x_{i}^{2}\right)$
Step	[−100, 100]	$f_{2}\left(\vec{x}\right)=\sum_{i=1}^{D}\left(x_{i}^{2}\right)$
Schwefel	[−10, 10]	$f_{3}\left(\vec{x}\right)=\sum_{i=1}^{D}\left(-x_{i}\sin\left(\sqrt{\left|x_{i}\right|}\right)\right)$
Rosenbrock valley	[−30, 30]	$f_{4}\left(\vec{x}\right)=\sum_{i=1}^{D-1}\left(100{\left(x_{i+1}-x_{i}^{2}\right)}^{2}+{\left(x_{i}-1\right)}^{2}\right)$
Dixon-Price	[−10, 10]	$f_{5}\left(\vec{x}\right)={\left(x_{1}-1\right)}^{2}+\sum_{i=2}^{D}\left(i{\left(2x_{i}^{2}-x_{i-1}\right)}^{2}\right)$
Rastrigin	[−5.12, 5.12]	$f_{6}\left(\vec{x}\right)=\sum_{i=1}^{D}\left(x_{i}^{2}-10\;\cos\left(2\pi x_{i}\right)+10\right)$
Griewank	[−600, 600]	$f_{7}\left(\vec{x}\right)=1/4000\left(\sum_{i=1}^{D}x_{i}^{2},\right)-\left(\prod_{i=1}^{D}\cos\left(x_{i}/\sqrt{i}\right)\right)+1$
Ackley	[−10, 10]	$f_{8}\left(\vec{x}\right)=20+e-20\;\;\exp\left(-0.2\sqrt{\left(1/D\right)\sum_{i=1}^{D}x_{i}^{2}}\right)-\exp\left(\left(1/D\right)\sum_{i=1}^{D}\cos\left(2\pi x_{i}\right)\right)$
Penalized	[−30, 30]	$\begin{array}{c} f_{9}\left(\vec{x}\right)=\left(\pi /D\right)\left(10\;\sin^{2}\left(\pi y_{i}\right)+\left(\sum_{i=1}^{D}{\left(y_{i}-1\right)}^{2}\left(1+10\;\sin^{2}\left(\pi y_{i+1}\right)\right)\right)\right)\;+\;\sum_{i=1}^{D}u\left(x_{i},10,100,4\right)\\ u\left(x_{i},a,k,m\right)=\left\{\begin{array}{cc} k{\left(x_{i}-a\right)}^{m}, & x_{i}>a,\\ 0, & -a\leq x_{i}\leq a,\\ k{\left(-x_{i}-a\right)}^{m}, & x_{i}, \end{array}\right\}y_{i}=1+\left(1/4\right)\left(x_{i}+1\right) \end{array}$

**Table 2:** Results of the ABC algorithms on 10-dimensional functions for 100, 000 evaluations.

Function		ABC	tlABC (tf=0.25)	tlABC (tf=0.50)	tlABC (tf=0.75)	tlABC (tf=1.00)
*f*_1_	Mean	5.693663*e* − 45	**2.953714*e* − 45**	6.269275*e* − 45	6.530371*e* − 45	3.273292*e* − 42
	Std. dev.	8.439450*e* − 45	5.063626*e* − 45	9.249126*e* − 45	9.447314*e* − 45	9.106988*e* − 42

*f*_2_	Mean	**0.000000*e* + 00**	**0.000000*e* + 00**	**0.000000*e* + 00**	**0.000000*e* + 00**	**0.000000*e* + 00**
	Std. dev.	0.000000*e* + 00	0.000000*e* + 00	0.000000*e* + 00	0.000000*e* + 00	0.000000*e* + 00

*f*_3_	Mean	**−4.199780*e* + 03**	**−4.199780*e* + 03**	**−4.199780*e* + 03**	**−4.199780*e* + 03**	**−4.199780*e* + 03**
	Std. dev.	0.000000*e* + 00	0.000000*e* + 00	0.000000*e* + 00	0.000000*e* + 00	0.000000*e* + 00

*f*_4_	Mean	2.979121*e* − 01	3.778179*e* − 01	**1.742658*e* − 01**	4.115360*e* − 01	4.038612*e* − 01
	Std. dev.	4.223787*e* − 01	5.313373*e* − 01	3.892949*e* − 01	8.611120*e* − 01	4.975071*e* − 01

*f*_5_	Mean	1.826027*e* − 06	1.894760*e* − 06	1.713676*e* − 06	5.825965*e* − 07	**3.601930*e* − 07**
	Std. dev.	1.883324*e* − 06	1.430275*e* − 06	4.165584*e* − 06	7.745276*e* − 07	3.184672*e* − 07

*f*_6_	Mean	**0.000000*e* + 00**	**0.000000*e* + 00**	**0.000000*e* + 00**	**0.000000*e* + 00**	**0.000000*e* + 00**
	Std. dev.	0.000000*e* + 00	0.000000*e* + 00	0.000000*e* + 00	0.000000*e* + 00	0.000000*e* + 00

*f*_7_	Mean	**0.000000*e* + 00**	**0.000000*e* + 00**	**0.000000*e* + 00**	**0.000000*e* + 00**	**0.000000*e* + 00**
	Std. dev.	0.000000*e* + 00	0.000000*e* + 00	0.000000*e* + 00	0.000000*e* + 00	0.000000*e* + 00

*f*_8_	Mean	**4.440892*e* − 15**	**4.440892*e* − 15**	**4.440892*e* − 15**	**4.440892*e* − 15**	**4.440892*e* − 15**
	Std. dev.	2.407040*e* − 30	2.407040*e* − 30	2.407040*e* − 30	2.407040*e* − 30	2.407040*e* − 30

*f*_9_	Mean	**4.711634*e* − 32**	**4.711634*e* − 32**	**4.711634*e* − 32**	**4.711634*e* − 32**	**4.711634*e* − 32**
	Std. dev.	1.113480*e* − 47	1.113480*e* − 47	1.113480*e* − 47	1.113480*e* − 47	1.113480*e* − 47

**Table 3:** Results of the ABC algorithms on 100-dimensional functions for 100, 000 evaluations.

Function		ABC	tlABC (tf=0.25)	tlABC (tf=0.50)	tlABC (tf=0.75)	tlABC (tf=1.00)
*f*_1_	Mean	**1.146230*e* − 04**	9.116041*e* − 02	2.180088*e* − 03	4.035060*e* − 02	3.389584*e* − 04
	Std. dev.	5.378291*e* − 05	4.907924*e* − 01	1.138153*e* − 02	2.203417*e* − 01	5.815809*e* − 04

*f*_2_	Mean	**0.000000*e* + 00**	**0.000000*e* + 00**	**0.000000*e* + 00**	**0.000000*e* + 00**	**0.000000*e* + 00**
	Std. dev.	0.000000*e* + 00	0.000000*e* + 00	0.000000*e* + 00	0.000000*e* + 00	0.000000*e* + 00

*f*_3_	Mean	−3.503712*e* + 04	−3.512345*e* + 04	−3.506332*e* + 04	−3.549973*e* + 04	**−3.796363*e* + 04**
	Std. dev.	4.481348*e* + 02	3.707149*e* + 02	4.999285*e* + 02	4.174149*e* + 02	4.526192*e* + 02

*f*_4_	Mean	1.067490*e* + 03	**7.545328*e* + 02**	1.527744*e* + 03	1.411030*e* + 03	1.081109*e* + 03
	Std. dev.	2.212599*e* + 03	1.943518*e* + 03	3.214016*e* + 03	2.806875*e* + 03	2.450082*e* + 03

*f*_5_	Mean	1.858181*e* + 01	1.721964*e* + 01	**1.230956*e* + 01**	1.666567*e* + 01	2.198629*e* + 01
	Std. dev.	9.809400*e* + 00	1.337142*e* + 01	1.112425*e* + 01	8.603978*e* + 00	1.381023*e* + 01

*f*_6_	Mean	8.070312*e* + 01	7.770167*e* + 01	7.960197*e* + 01	7.353233*e* + 01	**2.657986*e* + 01**
	Std. dev.	8.297471*e* + 00	9.088505*e* + 00	8.882800*e* + 00	1.341344*e* + 01	8.194344*e* + 00

*f*_7_	Mean	5.623082*e* − 03	4.471904*e* − 03	1.521697*e* − 03	1.226925*e* − 03	**9.067452*e* − 04**
	Std. dev.	6.444604*e* − 03	7.222166*e* − 03	2.774665*e* − 03	3.971804*e* − 03	2.929140*e* − 03

*f*_8_	Mean	3.506393*e* + 00	3.376353*e* + 00	3.409211*e* + 00	3.334140*e* + 00	**6.163238*e* − 01**
	Std. dev.	2.741636*e* − 01	3.715616*e* − 01	3.869801*e* − 01	2.544070*e* − 01	4.534197*e* − 01

*f*_9_	Mean	1.430508*e* − 06	7.407323*e* − 07	**5.851995*e* − 07**	7.236565*e* − 07	1.866245*e* − 06
	Std. dev.	1.666006*e* − 06	5.350666*e* − 07	4.549148*e* − 07	5.976208*e* − 07	5.738448*e* − 06

**Table 4:** Results of the ABC algorithms on 500-dimensional functions for 100, 000 evaluations.

Function		ABC	tlABC (tf=0.25)	tlABC (tf=0.50)	tlABC (tf=0.75)	tlABC (tf=1.00)
*f*_1_	Mean	4.130324*e* + 05	4.081565*e* + 05	4.092916*e* + 05	4.148888*e* + 05	**1.765257*e* + 05**
	Std. dev.	2.657309*e* + 04	2.109512*e* + 04	3.163729*e* + 04	1.841777*e* + 04	3.476805*e* + 04

*f*_2_	Mean	4.141206*e* + 05	4.055300*e* + 05	4.100318*e* + 05	4.141699*e* + 05	**1.751431*e* + 05**
	Std. dev.	2.113762*e* + 04	1.980737*e* + 04	2.557841*e* + 04	2.526127*e* + 04	3.221499*e* + 04

*f*_3_	Mean	−1.095819*e* + 05	−1.104961*e* + 05	−1.115083*e* + 05	−1.255785*e* + 05	**−1.331409*e* + 05**
	Std. dev.	3.301774*e* + 03	2.736786*e* + 03	3.422581*e* + 03	4.460019*e* + 03	4.342221*e* + 03

*f*_4_	Mean	1.476707*e* + 11	1.438909*e* + 11	1.423152*e* + 11	1.357231*e* + 11	**2.494721*e* + 10**
	Std. dev.	1.758631*e* + 10	1.986122*e* + 10	2.000721*e* + 10	1.653048*e* + 10	1.555079*e* + 10

*f*_5_	Mean	1.312933*e* + 08	1.228235*e* + 08	1.297045*e* + 08	1.320696*e* + 08	**2.468820*e* + 07**
	Std. dev.	2.000876*e* + 07	2.804387*e* + 07	2.248323*e* + 07	1.988607*e* + 07	2.104472*e* + 07

*f*_6_	Mean	3.782787*e* + 03	3.779277*e* + 03	3.752828*e* + 03	3.806080*e* + 03	**2.648505*e* + 03**
	Std. dev.	8.469962*e* + 01	7.757594*e* + 01	8.311950*e* + 01	1.159574*e* + 02	1.674382*e* + 02

*f*_7_	Mean	3.716680*e* + 03	3.699020*e* + 03	3.720641*e* + 03	3.756675*e* + 03	**1.685117*e* + 03**
	Std. dev.	2.821720*e* + 02	2.246426*e* + 02	1.920369*e* + 02	2.167446*e* + 02	3.289570*e* + 02

*f*_8_	Mean	1.898080*e* + 01	1.899767*e* + 01	1.896413*e* + 01	1.898831*e* + 01	**1.741548*e* + 01**
	Std. dev.	1.015718*e* − 01	9.901172*e* − 02	9.528671*e* − 02	1.041574*e* − 01	3.073344*e* − 01

*f*_9_	Mean	2.220318*e* + 09	2.213338*e* + 09	2.164662*e* + 09	1.934285*e* + 09	**2.277159*e* + 08**
	Std. dev.	3.493875*e* + 08	3.934482*e* + 08	4.189587*e* + 08	3.976640*e* + 08	2.436363*e* + 08

**Table 5:** Results of the ABC algorithms on 10-dimensional functions for 1, 000, 000 evaluations.

Function		ABC	tlABC (tf=0.25)	tlABC (tf=0.50)	tlABC (tf=0.75)	tlABC (tf=1.00)
*f*_1_	Mean	**0.000000*e* + 00**	**0.000000*e* + 00**	**0.000000*e* + 00**	**0.000000*e* + 00**	**0.000000*e* + 00**
	Std. dev.	0.000000*e* + 00	0.000000*e* + 00	0.000000*e* + 00	0.000000*e* + 00	0.000000*e* + 00

*f*_2_	Mean	**0.000000*e* + 00**	**0.000000*e* + 00**	**0.000000*e* + 00**	**0.000000*e* + 00**	**0.000000*e* + 00**
	Std. dev.	0.000000*e* + 00	0.000000*e* + 00	0.000000*e* + 00	0.000000*e* + 00	0.000000*e* + 00

*f*_3_	Mean	**−4.199780*e* + 03**	**−4.199780*e* + 03**	**−4.199780*e* + 03**	**−4.199780*e* + 03**	**−4.199780*e* + 03**
	Std. dev.	0.000000*e* + 00	0.000000*e* + 00	0.000000*e* + 00	0.000000*e* + 00	0.000000*e* + 00

*f*_4_	Mean	1.287064*e* − 01	9.590407*e* − 02	**7.091962*e* − 02**	9.820524*e* − 02	9.099454*e* − 02
	Std. dev.	2.304770*e* − 01	1.964542*e* − 01	1.551120*e* − 01	1.479201*e* − 01	1.205863*e* − 01

*f*_5_	Mean	**2.189910*e* − 31**	2.255649*e* − 31	2.461081*e* − 31	2.354256*e* − 31	2.194019*e* − 31
	Std. dev.	4.596968*e* − 32	4.703618*e* − 32	5.507420*e* − 32	5.458371*e* − 32	4.684085*e* − 32

*f*_6_	Mean	**0.000000*e* + 00**	**0.000000*e* + 00**	**0.000000*e* + 00**	**0.000000*e* + 00**	**0.000000*e* + 00**
	Std. dev.	0.000000*e* + 00	0.000000*e* + 00	0.000000*e* + 00	0.000000*e* + 00	0.000000*e* + 00

*f*_7_	Mean	**0.000000*e* + 00**	**0.000000*e* + 00**	**0.000000*e* + 00**	**0.000000*e* + 00**	**0.000000*e* + 00**
	Std. dev.	0.000000*e* + 00	0.000000*e* + 00	0.000000*e* + 00	0.000000*e* + 00	0.000000*e* + 00

*f*_8_	Mean	2.190840*e* − 15	1.953992*e* − 15	1.480297*e* − 15	1.835569*e* − 15	**1.361874*e* − 15**
	Std. dev.	1.741300*e* − 15	1.655890*e* − 15	1.346653*e* − 15	1.597927*e* − 15	1.228336*e* − 15

*f*_9_	Mean	**4.711634*e* − 32**	**4.711634*e* − 32**	**4.711634*e* − 32**	**4.711634*e* − 32**	**4.711634*e* − 32**
	Std. dev.	1.113480*e* − 47	1.113480*e* − 47	1.113480*e* − 47	1.113480*e* − 47	1.113480*e* − 47

**Table 6:** Results of the ABC algorithms on 100-dimensional functions for 1, 000, 000 evaluations.

Function		ABC	tlABC (tf=0.25)	tlABC (tf=0.50)	tlABC (tf=0.75)	tlABC (tf=1.00)
*f*_1_	Mean	**6.006217*e* − 41**	8.496777*e* − 41	9.881287*e* − 41	1.697022*e* − 40	3.256242*e* − 40
	Std. dev.	5.222464*e* − 41	9.237328*e* − 41	1.105303*e* − 40	1.463845*e* − 40	8.193515*e* − 40

*f*_2_	Mean	**0.000000*e* + 00**	**0.000000*e* + 00**	**0.000000*e* + 00**	**0.000000*e* + 00**	**0.000000*e* + 00**
	Std. dev.	0.000000*e* + 00	0.000000*e* + 00	0.000000*e* + 00	0.000000*e* + 00	0.000000*e* + 00

*f*_3_	Mean	**−4.199726*e* + 04**	**−4.199730*e* + 04**	**−4.199732*e* + 04**	**−4.199728*e* + 04**	**−4.199764*e* + 04**
	Std. dev.	2.138699*e* − 01	2.477997*e* − 01	2.188478*e* − 01	2.229595*e* − 01	1.039474*e* − 01

*f*_4_	Mean	5.656192*e* − 01	4.949343*e* − 01	**3.653176*e* − 01**	5.381103*e* − 01	6.761292*e* − 01
	Std. dev.	1.648632*e* + 00	9.822533*e* − 01	6.077069*e* − 01	1.052070*e* + 00	1.032075*e* + 00

*f*_5_	Mean	3.390834*e* − 06	1.040101*e* − 06	2.820931*e* − 07	**9.399521*e* − 08**	1.349680*e* − 07
	Std. dev.	2.712035*e* − 06	1.051293*e* − 06	2.901874*e* − 07	1.104350*e* − 07	2.506659*e* − 07

*f*_6_	Mean	**0.000000*e* + 00**	**0.000000*e* + 00**	**0.000000*e* + 00**	**0.000000*e* + 00**	**0.000000*e* + 00**
	Std. dev.	0.000000*e* + 00	0.000000*e* + 00	0.000000*e* + 00	0.000000*e* + 00	0.000000*e* + 00

*f*_7_	Mean	**0.000000*e* + 00**	**0.000000*e* + 00**	**0.000000*e* + 00**	**0.000000*e* + 00**	**0.000000*e* + 00**
	Std. dev.	0.000000*e* + 00	0.000000*e* + 00	0.000000*e* + 00	0.000000*e* + 00	0.000000*e* + 00

*f*_8_	Mean	**4.056015*e* − 14**	**4.056015*e* − 14**	4.138911*e* − 14	4.174439*e* − 14	4.778400*e* − 14
	Std. dev.	1.346651*e* − 15	1.638272*e* − 15	1.770219*e* − 15	1.806722*e* − 15	3.285392*e* − 15

*f*_9_	Mean	**4.711634*e* − 33**	**4.711634*e* − 33**	**4.711634*e* − 33**	**4.711634*e* − 33**	**4.711634*e* − 33**
	Std. dev.	2.783699*e* − 48	2.783699*e* − 48	2.783699*e* − 48	2.783699*e* − 48	2.783699*e* − 48

**Table 7:** Results of the ABC algorithms on 500-dimensional functions for 1, 000, 000 evaluations.

Function		ABC	tlABC (tf=0.25)	tlABC (tf=0.50)	tlABC (tf=0.75)	tlABC (tf=1.00)
*f*_1_	Mean	1.290605*e* − 06	1.799212*e* − 07	1.883553*e* − 07	**1.104870*e* − 07**	9.142393*e* − 07
	Std. dev.	2.802508*e* − 06	1.406763*e* − 07	1.233807*e* − 07	1.051293*e* − 07	1.875214*e* − 06

*f*_2_	Mean	**0.000000*e* + 00**	**0.000000*e* + 00**	**0.000000*e* + 00**	**0.000000*e* + 00**	**0.000000*e* + 00**
	Std. dev.	0.000000*e* + 00	0.000000*e* + 00	0.000000*e* + 00	0.000000*e* + 00	0.000000*e* + 00

*f*_3_	Mean	−1.887655*e* + 05	−1.887683*e* + 05	−1.889353*e* + 05	−1.895722*e* + 05	**−1.988947*e* + 05**
	Std. dev.	7.730400*e* + 02	1.064341*e* + 03	7.961808*e* + 02	7.173520*e* + 02	1.389004*e* + 03

*f*_4_	Mean	9.185335*e* + 02	**6.244401*e* + 02**	1.233145*e* + 03	6.366118*e* + 02	1.144464*e* + 03
	Std. dev.	1.156629*e* + 03	1.692910*e* + 02	2.355193*e* + 03	2.280679*e* + 02	1.706124*e* + 03

*f*_5_	Mean	2.721353*e* + 02	**2.431451*e* + 02**	2.402067*e* + 02	2.651948*e* + 02	2.567815*e* + 02
	Std. dev.	9.054174*e* + 01	9.960557*e* + 01	8.497216*e* + 01	8.850773*e* + 01	1.086047*e* + 02

*f*_6_	Mean	1.390284*e* + 02	1.379240*e* + 02	1.413926*e* + 02	1.390184*e* + 02	**4.883904*e* + 01**
	Std. dev.	1.311659*e* + 01	1.110394*e* + 01	9.133143*e* + 00	1.336659*e* + 01	1.224055*e* + 01

*f*_7_	Mean	5.312618*e* − 06	9.069779*e* − 07	**1.961672*e* − 08**	3.092865*e* − 08	9.607941*e* − 08
	Std. dev.	1.306894*e* − 05	7.826006*e* − 07	2.556943*e* − 08	1.478953*e* − 07	5.036284*e* − 07

*f*_8_	Mean	3.834920*e* − 01	3.648266*e* − 01	3.758358*e* − 01	3.522330*e* − 01	**3.186149*e* − 03**
	Std. dev.	9.247263*e* − 02	9.051693*e* − 02	9.648746*e* − 02	8.698696*e* − 02	2.611740*e* − 03

*f*_9_	Mean	7.184301*e* − 09	1.678214*e* − 09	6.241791*e* − 10	**3.871350*e* − 10**	6.344426*e* − 10
	Std. dev.	2.688062*e* − 09	5.872811*e* − 09	6.111414*e* − 10	5.460230*e* − 10	1.619332*e* − 09

**Table 8:** Statistical comparison between ABC and tlABC algorithms for 1, 000, 000 evaluations.

Function	ABC-tlABC (tf=0.25)		ABC-tlABC (tf=0.50)		ABC-tlABC (tf=0.75)		ABC-tlABC (tf=1.00)	
	*p* value	Sign.	*p* value	Sign.	*p* value	Sign.	*p* value	Sign.
*f*_1_	0.000002	**tlABC**	0.000002	**tlABC**	0.000002	**tlABC**	0.018519	**tlABC**
*f*_2_	1.000000	—	1.000000	—	1.000000	—	1.000000	—
*f*_3_	0.797098	—	0.338856	—	0.001484	**tlABC**	0.000002	**tlABC**
*f*_4_	0.253644	—	0.490798	—	0.544006	—	0.019569	**ABC**
*f*_5_	0.198610	—	0.280214	—	0.614315	—	0.530440	—
*f*_6_	0.765519	—	0.280214	—	0.718888	—	0.000002	**tlABC**
*f*_7_	0.000020	**tlABC**	0.000002	**tlABC**	0.000002	**tlABC**	0.000002	**tlABC**
*f*_8_	0.557743	—	0.749871	—	0.198610	—	0.000002	**tlABC**
*f*_9_	0.000031	**tlABC**	0.000002	**tlABC**	0.000002	**tlABC**	0.000002	**tlABC**

**Table 9:** Statistical comparison between ABC and tlABC algorithms for 100, 000 evaluations.

Function	ABC-tlABC (tf=0.25)		ABC-tlABC (tf=0.50)		ABC-tlABC (tf=0.75)		ABC-tlABC (tf=1.00)	
	*p* value	Sign.	*p* value	Sign.	*p* value	Sign.	*p* value	Sign.
*f*_1_	0.643517	—	0.893644	—	0.571646	—	0.000002	**tlABC**
*f*_2_	0.165027	—	0.477947	—	0.765519	—	0.000002	**tlABC**
*f*_3_	0.289477	—	0.010444	**tlABC**	0.000002	**tlABC**	0.000002	**tlABC**
*f*_4_	0.360039	—	0.328571	—	0.008730	**tlABC**	0.000002	**tlABC**
*f*_5_	0.198610	—	0.718888	—	0.861213	—	0.000002	**tlABC**
*f*_6_	0.718888	—	0.165027	—	0.370935	—	0.000002	**tlABC**
*f*_7_	0.428430	—	0.503833	—	0.503833	—	0.000002	**tlABC**
*f*_8_	0.643517	—	0.289477	—	0.781264	—	0.000002	**tlABC**
*f*_9_	0.813017	—	0.599936	—	0.011748	—	0.000002	**tlABC**

**Table 10:** Success rates and mean fitness evaluations of the ABC and tlABC algorithms.

Function		ABC	tlABC (tf=0.25)	tlABC (tf=0.50)	tlABC (tf=0.75)	tlABC (tf=1.00)
*f*_1_	Sr	73.33	**100**	**100**	**100**	80
	Me	9.440909*e* + 05	8.661300*e* + 05	8.634300*e* + 05	**8.548233*e* + 05**	9.353458*e* + 05

*f*_2_	Sr	**100**	**100**	**100**	**100**	**100**
	Me	3.620033*e* + 05	3.573467*e* + 05	3.773100*e* + 05	3.319433*e* + 05	**2.342800*e* + 05**

*f*_3_	Sr	**100**	**100**	**100**	**100**	**100**
	Me	7.522333*e* + 04	7.410000*e* + 04	7.082667*e* + 04	4.963000*e* + 04	**4.227667*e* + 04**

*f*_4_	Sr	93.333333	**100**	96.66	**100**	80
	Me	4.938250*e* + 05	2.552267*e* + 05	2.555069*e* + 05	**2.526400*e* + 05**	6.011708*e* + 05

*f*_5_	Sr	**100**	**100**	**100**	**100**	**100**
	Me	2.583933*e* + 05	2.599000*e* + 05	2.681333*e* + 05	2.495200*e* + 05	**2.093467*e* + 05**

*f*_6_	Sr	0	0	0	0	**100**
	Me	—	—	—	—	**7.662900*e* + 05**

*f*_7_	Sr	0	80	**100**	**100**	96.66
	Me	—	8.865375*e* + 05	6.782433*e* + 05	**6.423900*e* + 05**	6.846966*e* + 05

*f*_8_	Sr	0	0	0	0	**100**
	Me	—	—	—	—	**8.905667*e* + 05**

*f*_9_	Sr	**100**	**100**	**100**	**100**	**100**
	Me	2.219033*e* + 05	2.083533*e* + 05	2.095567*e* + 05	1.912133*e* + 05	**1.484567*e* + 05**

**Table 11:** Results of the ABC variants and their tl-based implementations.

Function		GABC	tlGABC	ABC/best/1	tlABC/best/1	ABC/best/2	tlABC/best/2	CABC	tlCABC	EABC	tlEABC
*f*_1_	Mean	2.989080*e* + 05	**1.051314*e* + 05**	2.242907*e* + 05	**3.137669*e* + 04**	2.546813*e* + 05	**6.025662*e* + 04**	1.413895*e* + 05	**1.072005*e* + 03**	1.039612*e* + 05	**1.271344*e* + 04**
	Std.	6.937572*e* + 03	1.884551*e* + 04	9.762788*e* + 03	8.817089*e* + 03	1.407599*e* + 04	1.195514*e* + 04	2.301160*e* + 04	1.288588*e* + 03	1.010612*e* + 04	6.548458*e* + 03

*f*_2_	Mean	2.971081*e* + 05	**1.005788*e* + 05**	2.196111*e* + 05	**4.194133*e* + 04**	2.570600*e* + 05	**5.926310*e* + 04**	1.374307*e* + 05	**2.732100*e* + 03**	1.056779*e* + 05	**1.193113*e* + 04**
	Std.	1.549061*e* + 04	1.744929*e* + 04	8.855527*e* + 03	1.427391*e* + 04	1.246763*e* + 04	1.619039*e* + 04	2.767459*e* + 04	5.333241*e* + 03	1.128035*e* + 04	5.608269*e* + 03

*f*_3_	Mean	−1.105662*e* + 05	**−1.322008*e* + 05**	−1.173790*e* + 05	**−1.594298*e* + 05**	−1.134909*e* + 05	**−1.370600*e* + 05**	−1.221302*e* + 05	**−1.673471*e* + 05**	−1.059654*e* + 05	**−1.276223*e* + 05**
	Std.	1.667155*e* + 03	4.600184*e* + 03	1.733129*e* + 03	7.233451*e* + 03	1.809111*e* + 03	4.579026*e* + 03	2.511623*e* + 03	5.892463*e* + 03	2.110175*e* + 03	4.164446*e* + 03

*f*_4_	Mean	6.646385*e* + 08	**7.454488*e* + 07**	4.405736*e* + 08	**4.110608*e* + 06**	6.197777*e* + 10	**1.605348*e* + 09**	1.624091*e* + 10	**5.568477*e* + 05**	1.376731*e* + 10	**7.685256*e* + 04**
	Std.	8.548539*e* + 07	3.963909*e* + 07	6.114693*e* + 07	4.761904*e* + 06	4.610460*e* + 09	1.897109*e* + 09	1.240957*e* + 10	3.994781*e* + 05	6.373138*e* + 09	6.045023*e* + 04

*f*_5_	Mean	7.551259*e* + 07	**7.483358*e* + 06**	4.567554*e* + 07	**5.517947*e* + 05**	6.294965*e* + 07	**8.275073*e* + 05**	1.043841*e* + 07	**7.407381*e* + 03**	1.033071*e* + 07	**2.846864*e* + 03**
	Std.	8.872327*e* + 06	5.374782*e* + 06	6.532938*e* + 06	5.544704*e* + 05	8.307695*e* + 06	8.019661*e* + 05	1.054942*e* + 07	1.483032*e* + 03	6.374945*e* + 06	1.196192*e* + 03

*f*_6_	Mean	3.841937*e* + 03	**2.647297*e* + 03**	3.244925*e* + 03	**1.938399*e* + 03**	3.499391*e* + 03	**2.212203*e* + 03**	2.968246*e* + 03	**1.448452*e* + 03**	2.601508*e* + 03	**1.570249*e* + 03**
	Std.	1.024087*e* + 02	1.390625*e* + 02	5.549239*e* + 01	1.506975*e* + 02	6.663521*e* + 01	1.736415*e* + 02	9.608039*e* + 01	1.795641*e* + 02	4.681591*e* + 01	1.118736*e* + 02

*f*_7_	Mean	2.669574*e* + 03	**8.532467*e* + 02**	1.968510*e* + 03	**3.460704*e* + 02**	2.369774*e* + 03	**5.510468*e* + 02**	1.282921*e* + 03	**1.250417*e* + 01**	9.341722*e* + 02	**1.142156*e* + 02**
	Std.	1.265812*e* + 02	1.638093*e* + 02	9.198166*e* + 01	1.042930*e* + 02	1.041202*e* + 02	1.418946*e* + 02	1.797882*e* + 02	2.879077*e* + 01	7.628766*e* + 01	7.035969*e* + 01

*f*_8_	Mean	1.817634*e* + 01	**1.545382*e* + 01**	1.759370*e* + 01	**1.301224*e* + 01**	1.797928*e* + 01	**1.389394*e* + 01**	1.698323*e* + 01	**1.093645*e* + 01**	1.502982*e* + 01	**1.037044*e* + 01**
	Std.	9.915475*e* − 02	5.182776*e* − 01	1.049155*e* − 01	6.977710*e* − 01	1.022909*e* − 01	6.329151*e* − 01	1.800556*e* − 01	9.459496*e* − 01	1.962913*e* − 01	8.262012*e* − 01

*f*_9_	Mean	3.946945*e* + 07	**2.661506*e* + 05**	1.688291*e* + 07	**1.048118*e* − 01**	6.774404*e* + 08	**3.060992*e* + 05**	2.932435*e* + 07	**1.040208*e* − 01**	6.241820*e* + 07	**2.164166*e* − 02**
	Std.	1.204241*e* + 07	6.401036*e* + 05	7.795573*e* + 06	1.014036*e* − 01	2.189384*e* + 08	8.185503*e* + 05	6.243321*e* + 07	2.311059*e* − 02	8.520401*e* + 07	8.204890*e* − 03

**Table 12:** Statistical comparison between ABC variants and their tl-based implementations.

Function	GABC-tlGABC		ABC/b/1-tlABC/best/1		ABC/best/2-tlABC/b/2		CABC-tlCABC		EABC-tlEABC	
	*p* value	Sign.	*p* value	Sign.	*p* value	Sign.	*p* value	Sign.	*p* value	Sign.
*f*_1_	0.000002	**tlGABC**	0.000002	**tlABC/best/1**	0.000002	**tlABC/best/2**	0.000002	**tlCABC**	0.000002	**tlEABC**
*f*_2_	0.000002	**tlGABC**	0.000002	**tlABC/best/1**	0.000002	**tlABC/best/2**	0.000002	**tlCABC**	0.000002	**tlEABC**
*f*_3_	0.000002	**tlGABC**	0.000002	**tlABC/best/1**	0.000002	**tlABC/best/2**	0.000002	**tlCABC**	0.000002	**tlEABC**
*f*_4_	0.000002	**tlGABC**	0.000002	**tlABC/best/1**	0.000002	**tlABC/best/2**	0.000002	**tlCABC**	0.000002	**tlEABC**
*f*_5_	0.000002	**tlGABC**	0.000002	**tlABC/best/1**	0.000002	**tlABC/best/2**	0.000002	**tlCABC**	0.000002	**tlEABC**
*f*_6_	0.000002	**tlGABC**	0.000002	**tlABC/best/1**	0.000002	**tlABC/best/2**	0.000002	**tlCABC**	0.000002	**tlEABC**
*f*_7_	0.000002	**tlGABC**	0.000002	**tlABC/best/1**	0.000002	**tlABC/best/2**	0.000002	**tlCABC**	0.000002	**tlEABC**
*f*_8_	0.000002	**tlGABC**	0.000002	**tlABC/best/1**	0.000002	**tlABC/best/2**	0.000002	**tlCABC**	0.000002	**tlEABC**
*f*_9_	0.000002	**tlGABC**	0.000002	**tlABC/best/1**	0.000002	**tlABC/best/2**	0.000002	**tlCABC**	0.000002	**tlEABC**

**Table 13:** Results of the serial and parallel ABC algorithms for 200, 000 evaluations.

Function		ABC	p-ABC	tlABC (tf=1.00)	p-tlABC (tf=1.00)
*f*_1_	Mean	3.910855*e* + 05	4.113880*e* + 05	**1.511925*e* + 05**	2.623364*e* + 05
	Std. dev.	2.250835*e* + 04	2.045026*e* + 04	3.217609*e* + 04	2.342807*e* + 04

*f*_2_	Mean	3.952807*e* + 05	4.063917*e* + 05	**1.703726*e* + 05**	2.542833*e* + 05
	Std. dev.	2.168618*e* + 04	2.252281*e* + 04	2.670481*e* + 04	1.531853*e* + 04

*f*_3_	Mean	−1.107573*e* + 05	−1.060833*e* + 05	**−1.337285*e* + 05**	−1.153257*e* + 05
	Std. dev.	2.056823*e* + 03	1.688084*e* + 03	3.200059*e* + 03	2.056774*e* + 0

*f*_4_	Mean	1.313768*e* + 11	1.410091*e* + 11	**2.129992*e* + 10**	7.021863*e* + 10
	Std. dev.	1.563876*e* + 10	1.599200*e* + 10	1.582424*e* + 10	1.513810*e* + 10

*f*_5_	Mean	1.237054*e* + 08	1.292475*e* + 08	**1.328077*e* + 07**	6.451395*e* + 07
	Std. dev.	1.583419*e* + 07	1.709901*e* + 07	1.146906*e* + 07	1.386868*e* + 07

*f*_6_	Mean	3.729256*e* + 03	3.704902*e* + 03	**2.616509*e* + 03**	3.034230*e* + 03
	Std. dev.	8.960394*e* + 01	9.183761*e* + 01	1.120117*e* + 02	6.872550*e* + 01

*f*_7_	Mean	3.596772*e* + 03	3.673797*e* + 03	**1.449607*e* + 03**	2.258571*e* + 03
	Std. dev.	2.262908*e* + 02	1.946942*e* + 02	3.173375*e* + 02	1.513701*e* + 02

*f*_8_	Mean	1.892315*e* + 01	1.886268*e* + 01	**1.719341*e* + 01**	1.793002*e* + 01
	Std. dev.	8.614159*e* − 02	1.102155*e* − 01	3.049814*e* − 01	1.375368*e* − 01

*f*_9_	Mean	1.928259*e* + 09	2.055529*e* + 09	**4.621482*e* + 07**	8.822927*e* + 08
	Std. dev.	4.279168*e* + 08	3.681661*e* + 08	8.184589*e* + 07	2.899285*e* + 08

**Table 14:** Results of the serial and parallel ABC algorithms for 500, 000 evaluations.

Function		ABC	p-ABC	tlABC (tf=1.00)	p-tlABC (tf=1.00)
*f*_1_	Mean	1.826096*e* + 04	5.033667*e* + 04	**8.409015*e* − 01**	1.956916*e* + 04
	Std. dev.	8.633110*e* + 03	7.271991*e* + 03	3.655900*e* + 00	7.453569*e* + 03

*f*_2_	Mean	2.257667*e* + 04	5.040037*e* + 04	**0.000000*e* + 00**	1.969877*e* + 04
	Std. dev.	6.660677*e* + 03	8.124214*e* + 03	0.000000*e* + 00	6.797547*e* + 03

*f*_3_	Mean	−1.443385*e* + 05	−1.402345*e* + 05	**−1.682593*e* + 05**	−1.488086*e* + 05
	Std. dev.	1.613792*e* + 03	1.521322*e* + 03	2.061252*e* + 03	2.732394*e* + 03

*f*_4_	Mean	7.253052*e* + 06	8.656352*e* + 09	**2.262458*e* + 03**	5.254016*e* + 09
	Std. dev.	3.938622*e* + 07	4.774166*e* + 09	2.536171*e* + 03	3.870297*e* + 09

*f*_5_	Mean	6.117529*e* + 02	1.029120*e* + 07	**4.049498*e* + 02**	6.014724*e* + 06
	Std. dev.	1.653580*e* + 02	4.945648*e* + 06	2.878103*e* + 02	3.192987*e* + 06

*f*_6_	Mean	1.501114*e* + 03	1.600520*e* + 03	**8.280762*e* + 02**	1.166655*e* + 03
	Std. dev.	4.089167*e* + 01	6.851913*e* + 01	5.907564*e* + 01	6.468204*e* + 01

*f*_7_	Mean	1.441019*e* + 02	4.398386*e* + 02	**5.415552*e* − 02**	1.930690*e* + 02
	Std. dev.	7.056074*e* + 01	7.253617*e* + 01	1.388155*e* − 01	8.685482*e* + 01

*f*_8_	Mean	1.328369*e* + 01	1.373531*e* + 01	**7.759852*e* + 00**	1.087339*e* + 01
	Std. dev.	2.809899*e* − 01	3.160843*e* − 01	5.969437*e* − 01	5.216135*e* − 01

*f*_9_	Mean	1.397777*e* − 05	1.322906*e* + 08	**4.854342*e* − 06**	7.925567*e* + 07
	Std. dev.	1.082536*e* − 05	8.511302*e* + 07	1.150941*e* − 06	6.522480*e* + 07

**Table 15:** Statistical comparison between serial and parallel ABC algorithms for 200, 000 evaluations.

Function	ABC vs p-tlABC (tf=1.00)		p-ABC vs p-tlABC (tf=1.00)		tlABC (tf=1.00) vs p-tlABC (tf=1.00)	
	*p* value	Sign.	*p* value	Sign.	*p* value	Sign.
*f*_1_	0.000002	**p-tlABC**	0.000002	**p-tlABC**	0.000002	**tlABC**
*f*_2_	0.000002	**p-tlABC**	0.000002	**p-tlABC**	0.000002	**tlABC**
*f*_3_	0.000003	**p-tlABC**	0.000002	**p-tlABC**	0.000002	**tlABC**
*f*_4_	0.000002	**p-tlABC**	0.000002	**p-tlABC**	0.000002	**tlABC**
*f*_5_	0.000002	**p-tlABC**	0.000002	**p-tlABC**	0.000002	**tlABC**
*f*_6_	0.000002	**p-tlABC**	0.000002	**p-tlABC**	0.000002	**tlABC**
*f*_7_	0.000002	**p-tlABC**	0.000002	**p-tlABC**	0.000002	**tlABC**
*f*_8_	0.000002	**p-tlABC**	0.000002	**p-tlABC**	0.000002	**tlABC**
*f*_9_	0.000002	**p-tlABC**	0.000002	**p-tlABC**	0.000002	**tlABC**

**Table 16:** Statistical comparison between serial and parallel ABC algorithms for 500, 000 evaluations.

Function	ABC vs p-tlABC (tf=1.00)		p-ABC vs p-tlABC (tf=1.00)		tlABC (tf=1.00) vs p-tlABC (tf=1.00)	
	*p* value	Sign.	*p* value	Sign.	*p* value	Sign.
*f*_1_	0.628843	—	0.000002	**p-tlABC**	0.000002	**tlABC**
*f*_2_	0.102011	—	0.000002	**p-tlABC**	0.000002	**tlABC**
*f*_3_	0.000006	**p-tlABC**	0.000002	**p-tlABC**	0.000002	**tlABC**
*f*_4_	0.000002	**ABC**	0.000063	**p-tlABC**	0.000002	**tlABC**
*f*_5_	0.000002	**ABC**	0.000097	**p-tlABC**	0.000002	**tlABC**
*f*_6_	0.000002	**p-tlABC**	0.000002	**p-tlABC**	0.000002	**tlABC**
*f*_7_	0.015658	**ABC**	0.000002	**p-tlABC**	0.000002	**tlABC**
*f*_8_	0.000002	**p-tlABC**	0.000002	**p-tlABC**	0.000002	**tlABC**
*f*_9_	0.000002	**ABC**	0.019569	**p-tlABC**	0.000002	**tlABC**

**Table 17:** Average execution times for ABC algorithms for 200, 000 evaluations.

Function		ABC	p-ABC	tlABC (tf=1.00)	p-tlABC (tf=1.00)
*f*_1_	Mean	1.477644	0.866410	1.087281	0.798863
	Std. dev.	5.720402*e* − 03	4.747840*e* − 02	3.302129*e* − 02	4.743118*e* − 02

*f*_2_	Mean	3.607676	1.126180	2.965352	1.053342
	Std. dev.	6.229590*e* − 02	5.275899*e* − 02	8.757225*e* − 02	4.813870*e* − 02

*f*_3_	Mean	8.162889	2.365428	7.180223	2.267182
	Std. dev.	4.064324*e* − 01	2.225126*e* − 01	1.890232*e* − 02	9.763186*e* − 02

*f*_4_	Mean	2.757862	0.974841	2.437673	0.923312
	Std. dev.	8.168550*e* − 02	4.322536*e* − 02	2.583628*e* − 02	5.258972*e* − 02

*f*_5_	Mean	5.164712	1.550928	4.149877	1.576741
	Std. dev.	3.076150*e* − 01	6.867118*e* − 02	1.758990*e* − 01	9.186976*e* − 02

*f*_6_	Mean	6.925959	1.872074	6.645661	2.070032
	Std. dev.	2.244740*e* − 01	8.358303*e* − 02	7.833041*e* − 02	1.565193*e* − 01

*f*_7_	Mean	14.850998	5.088426	14.155656	5.085120
	Std. dev.	6.641737*e* − 02	6.383821*e* − 01	1.274310*e* − 01	5.982128*e* − 01

*f*_8_	Mean	8.294480	2.537991	8.270296	2.664046
	Std. dev.	4.934985*e* − 01	1.996940*e* − 01	3.219023*e* − 02	3.379277*e* − 01

*f*_9_	Mean	19.338706	7.723584	18.418161	7.223549
	Std. dev.	2.137728*e* − 01	1.025024*e* + 00	1.667562*e* − 01	1.167932*e* + 00

**Table 18:** Average execution times for ABC algorithms for 500, 000 evaluations.

Function		ABC	p-ABC	tlABC (tf=1.00)	p-tlABC (tf=1.00)
*f*_1_	Mean	3.750697	2.237923	2.736264	2.046743
	Std. dev.	1.452007*e* − 02	1.226359*e* − 01	8.310174*e* − 02	1.215219*e* − 01

*f*_2_	Mean	9.256972	2.915909	7.893101	2.871335
	Std. dev.	1.598457*e* − 01	1.366038*e* − 01	2.330977*e* − 01	1.312227*e* − 01

*f*_3_	Mean	21.025269	6.374496	19.874767	6.265583
	Std. dev.	1.046854*e* + 00	5.996401*e* − 01	5.232140*e* − 02	2.698154*e* − 01

*f*_4_	Mean	7.578933	2.513816	6.203705	2.477468
	Std. dev.	2.244815*e* − 01	1.114650*e* − 01	6.575150*e* − 02	1.411108*e* − 01

*f*_5_	Mean	12.976374	4.107876	11.483484	4.201704
	Std. dev.	7.728847*e* − 01	1.818864*e* − 01	4.867455*e* − 01	2.448148*e* − 01

*f*_6_	Mean	17.730652	5.094427	16.961526	5.453327
	Std. dev.	5.746598*e* − 01	2.274524*e* − 01	1.999204*e* − 01	4.123372*e* − 01

*f*_7_	Mean	39.306712	13.101851	36.686701	13.032504
	Std. dev.	1.757894*e* − 01	1.643728*e* + 00	3.302583*e* − 01	1.533142*e* + 00

*f*_8_	Mean	22.354618	6.571634	21.359891	7.268796
	Std. dev.	1.330038*e* + 00	5.170688*e* − 01	8.313849*e* − 02	9.220289*e* − 01

*f*_9_	Mean	53.017629	48.477193	49.387006	19.334184
	Std. dev.	5.860643*e* − 01	4.389076*e* − 01	2.572918*e* + 00	3.126027*e* + 00

**Table 19:** Speedup and efficiency values for 200, 000 evaluations.

Function	ABC vs p-ABC		tlABC (tf=1.00) vs p-tlABC (tf=1.00)	
	Speedup	Efficiency	Speedup	Efficiency
*f*_1_	1.7055	0.4264	1.3610	0.3403
*f*_2_	3.2035	0.8009	2.8152	0.7038
*f*_3_	3.4509	0.8627	3.1670	0.7917
*f*_4_	2.8290	0.7073	2.6401	0.6600
*f*_5_	3.3301	0.8325	2.6319	0.6580
*f*_6_	3.6996	0.9249	3.2104	0.8026
*f*_7_	2.9186	0.7297	2.7837	0.6959
*f*_8_	3.2681	0.8170	3.1044	0.7761
*f*_9_	2.5039	0.6260	2.5497	0.6374
Average	2.9899	0.7475	2.6959	0.6740

**Table 20:** Speedup and efficiency values for 500,000 evaluations.

Function	ABC vs p-ABC		tlABC (tf=1.00) vs p-tlABC (tf=1.00)	
	Speedup	Efficiency	Speedup	Efficiency
*f*_1_	1.6760	0.4190	1.3369	0.3342
*f*_2_	3.1746	0.7936	2.7489	0.6872
*f*_3_	3.2983	0.8246	3.1721	0.7930
*f*_4_	3.0149	0.7537	2.5041	0.6260
*f*_5_	3.1589	0.7897	2.7331	0.6833
*f*_6_	3.4804	0.8701	3.1103	0.7776
*f*_7_	3.0001	0.7500	2.8150	0.7037
*f*_8_	3.4017	0.8504	2.9386	0.7347
*f*_9_	2.8694	0.7174	2.5544	0.6386
Average	3.0083	0.7521	2.6570	0.6643

**Table 21:** Best, average, and worst coverage values of ABC and tlABC algorithms.

Evaluations		ABC	p-ABC	tlABC (tf=1.00)	p-tlABC (tf=1.00)
2,000	Mean	0.906058	0.905176	**0.923302**	0.916322
	Best	0.914224	0.916479	0.935300	0.929713
	Worst	0.894520	0.897951	0.914714	0.904813
	Std. dev.	0.005607	0.005717	0.005741	0.005531

10,000	Mean	0.957019	0.963969	**0.969317**	0.957019
	Best	0.965690	0.967454	0.974610	0.965690
	Worst	0.944025	0.960494	0.963925	0.944025
	Std. dev.	0.006935	0.002131	0.002799	0.006935

**Table 22:** Average execution times of serial and parallel ABC algorithms.

Evaluations		ABC vs p-ABC		tlABC (tf=1.00) vs tl-p-ABC (tf=1.00)	
2,000	Mean	59.812201	33.713884	58.826034	31.508770
	Std. dev.	1.065064*e* + 00	3.546077*e* + 00	9.213946*e* − 01	2.380355*e* + 00

10,000	Mean	284.246941	168.547035	284.391910	167.229037
	Std. dev.	4.458504*e* + 00	7.173966*e* + 00	6.068226*e* + 00	7.173966*e* + 00

## Data Availability

No data were used to support this study.
